# *G. vaginalis* increases HSV-2 infection by decreasing vaginal barrier integrity and increasing inflammation *in vivo*

**DOI:** 10.3389/fimmu.2024.1487726

**Published:** 2024-11-22

**Authors:** Nuzhat Rahman, M. Firoz Mian, Christina L. Hayes, Aisha Nazli, Charu Kaushic

**Affiliations:** ^1^ Department of Medicine, McMaster University, Hamilton, ON, Canada; ^2^ McMaster Immunology Research Center, Michael G. DeGroote Center for Learning and Discovery, McMaster University, Hamilton, ON, Canada

**Keywords:** vaginal microbiota (VMB), *Lactobacillus*, bacterial vaginosis, herpes simplex virus, inflammation, barrier integrity, mouse models, female reproductive health

## Abstract

**Introduction:**

Clinically, a dysbiotic vaginal microbiota (VMB) colonized with anaerobic species such as *Gardnerella vaginalis* has been linked to increased susceptibility to viral sexually transmitted infections (STIs) such as Herpes Simplex Virus Type 2 (HSV-2). The mechanism is poorly understood due to the lack of small animal models.

**Methods:**

Mice were inoculated with 10^7^ CFU of the eubiotic bacteria *Lactobacillus crispatus*, the dysbiotic bacteria *G. vaginalis*, or PBS as a negative control every 48 h for ten days. On day ten, mice were inoculated with 10^5^ PFU WT HSV-2 333 and survival, pathology, and viral titers were assessed. To elucidate changes in the vaginal microenvironment following bacterial inoculations, vaginal tissue and washes were collected following ten days of inoculations. To assess barrier integrity, tissue was fixed and stained for the barrier protein Desmoglein-1 (DSG-1). To evaluate the immune microenvironment, tissue was processed for flow cytometry to examine tissue-resident T cells and cytokine production by T cells. Vaginal washes were used for multiplex cytokine/chemokine analysis.

**Results:**

*G. vaginalis* inoculated mice infected with HSV-2 had significantly decreased survival rates, increased pathology, and higher viral titers than PBS and *L. crispatus* inoculated mice. The vaginal epithelium of *G. vaginalis* inoculated mice showed decreased DSG-1 staining compared to other groups, indicating compromised barrier function. Decreased total numbers of CD4+ and CD8+ T cells expressing activated mucosal immune markers CD44, CD69, and CD103 were observed in the vaginal tract of *G. vaginalis* inoculated mice. They also showed increased proportions of T cells expressing inflammatory cytokines TNF-α and IFN-γ, while *L. crispatus* inoculated mice had increased proportions and absolute counts of T cells expressing the regulatory cytokine IL-10. In the multiplex assay, vaginal washes from *G. vaginalis* mice had increased inflammatory cytokines and chemokines compared to *L. crispatus* and PBS groups.

**Discussion:**

These results suggest *G. vaginalis* inoculation may be increasing HSV-2 infection by disrupting the epithelial barrier, decreasing protective immune responses and increasing tissue inflammation in the vaginal tract.

## Introduction

The female genital tract is colonized by an endogenous collection of microbes, termed the vaginal microbiota (VMB), which plays a critical role in female reproductive health (1). Studies have characterized a eubiotic VMB to be composed of *Lactobacillus* species, with *Lactobacillus crispatus* as the dominant species correlated with positive reproductive health outcomes and decreased risk of sexually transmitted infections (STIs) (2–4). A dysbiotic VMB is distinguished by a diverse community structure comprised of anaerobic bacteria such as *Gardnerella vaginalis*, a bacterial species that is commonly seen in individuals with the clinical condition bacterial vaginosis (BV) (2). A diverse VMB composed of various anaerobes is characteristic of dysbiosis, but as *G. vaginalis* is the most common bacteria seen in BV patients (5), it is the bacteria that is focused on in this study. Crucially, women with BV are at an increased risk of acquiring STIs. It is essential to understand the underlying mechanisms to design prevention strategies for at-risk populations.

Herpes Simplex Virus Type 2 (HSV-2) is a viral STI that poses a massive global health burden (6). In 2016, an estimated 491.5 million (13%) people aged 15–49 years were infected with HSV-2, with the highest number of new infections in adolescent women (6, 7). Heterosexual transmission is more efficient from men to women, where one study found HSV-2 is transmitted to women almost twice as often as to men (8). While studies have shown that behavioural factors such as the use of progestin-based contraceptives (9), having multiple sexual partners (10), and douching (11) can enhance HSV-2 risk, it is important to study biological factors contributing to why women are more susceptible to prevent the spread of new infections to vulnerable populations.

As previously mentioned, the distinct VMB of the female reproductive tract has been shown to play a role in increased susceptibility to STIs in women. There is a great deal of clinical and epidemiological evidence to support this claim (12–14). One group showed that a high Nugent score, typically seen in BV, is associated with a 2-fold increased risk of HSV-2 infection (12). In another study, the enrichment of dysbiotic species *G. vaginalis* and *Lactobacillus iners* was associated with an increased likelihood of HSV-2 in both male and female sexual partners (13). This same group also found that there are increased HSV-2 lesions and ulcers in BV-positive women (14). Overall, this clinical evidence suggests a dysbiotic VMB is linked to increased susceptibility to HSV-2.

The exact physiological mechanisms of how eubiotic and dysbiotic VMB alter the vaginal microenvironment are unclear. An *L. crispatus* dominant VMB may decrease susceptibility to STIs by decreasing vaginal pH through the secretion of hydrogen peroxide and acid metabolites such as lactic acid (2, 15). *G. vaginalis* may increase susceptibility to HSV-2 by reducing the mucus barrier (16), decreasing epithelial barrier integrity (17), and reducing immune function in the vaginal mucosa (18). However, while *in vitro* and clinical studies can provide correlative data, it is difficult to elucidate cause-effect mechanisms due to the lack of human VMB-associated mouse models. Recent *in vivo* models that harbour eubiotic and dysbiotic VMB provide a physiological system to experimentally elucidate how VMB composition may lead to changes in the vaginal mucosa, and the outcome this has on STI infections (19, 20). In the context of Human Immunodeficiency Virus (HIV), dysbiotic bacteria were found to increase the number of activated CD4+ cells in the female genital tract of germ-free mice, which are the primary target cells of HIV (21). Similar correlations to explain the increased susceptibility to HSV-2 in the context of a dysbiotic VMB have not been well investigated *in vivo*.

We have recently published a study describing a VMB-associated mouse model temporarily colonized with the eubiotic bacteria *L. crispatus* and the dysbiotic bacteria *G. vaginalis* (19). In this study, we found estrogen aided in the successful colonization of mice with eubiotic and dysbiotic species, potentially through a glycogen-mediated mechanism (19). This model was utilized in the current study to assess the role of the VMB on HSV-2 susceptibility *in vivo*. We found dysbiotic *G. vaginalis* inoculation to decrease survival rates and increase HSV-2 pathology and viral titers compared to the eubiotic bacteria *L. crispatus* and the PBS-negative control groups, validating that our model recapitulates clinical observations. As previously mentioned, two postulated mechanisms as to how *G. vaginalis* may increase susceptibility to HSV-2 are by disrupting the epithelial barrier and by altering the immune microenvironment in the vaginal tract. In this study, *G. vaginalis* decreased the expression of Desmoglein-1 (DSG-1), a junctional protein that binds vaginal epithelial cells together (22, 23). This indicates a disruption in the epithelium, which may be associated with the increased susceptibility to HSV-2. *G. vaginalis* was also associated with decreased mucosal resident T cells and an inflammatory signature, which can also be linked to less protective immune responses and increased pathology in the vaginal mucosa, leading to lower survival rates in *G. vaginalis* inoculated mice. Using our novel VMB-associated mouse models, this study demonstrates for the first time cause-effect mechanisms as to how a dysbiotic VMB may increase susceptibility to HSV-2.

## Materials and methods

### Mice

8-10-week-old female conventional C57BL/6 mice were obtained from Charles River Laboratories (Saint-Constant, QC, Canada). All mice were maintained under specific pathogen-free and standard temperature-controlled conditions that followed a 12-hour light/dark cycle at the Central Animal Facility at McMaster University. Mice were allowed one week after arrival to acclimate prior to experimental use. All mouse studies performed were approved by and complied with the Animal Research Ethics Board (AREB) at McMaster University (AUP 22-03-07).

### Colonization of mice

Bacteria were prepared as per previously published protocols (19). Briefly, *Lactobacillus crispatus* SJ-3C-US was grown in American Type Culture Collection (ATCC) medium 416 (*Lactobacillus* MRS broth) at 37°C in anaerobic conditions for 24 hours. *Gardnerella vaginalis* ATCC 14019 was grown in ATCC medium 1685 (NYC III medium) at 37°C in anaerobic conditions for 24 hours. Bacteria were then washed once with Phosphate-buffered saline (PBS) and resuspended in 25 µL PBS at 10^7^ colony-forming units (CFU) per mouse. To colonize the mice, mice were inoculated intravaginally with 25 µL of the bacteria and then held facedown for one minute to allow for the bacteria to persist in the vaginal canal. To evaluate colonization, vaginal washes were collected from the mice and quantitative plating assays were performed as described previously using the Miles and Misra technique (19, 24). To maintain consistent colonization in the mice, *L. crispatus* and *G. vaginalis* were inoculated every 48 hours for ten days.

### Estrus cycle staging

10 µL of vaginal washes were pipetted onto a glass slide and viewed under a microscope. The cells were observed and compared to images of vaginal washes from mice under different stages of the estrus cycle (25). Briefly, vaginal washes from mice in the estrus stage of their cycle were primarily composed of keratinized epithelial cells and vaginal washes from mice in the diestrus stage were composed primarily of leukocytes.

### HSV-2 infection in bacteria colonized mice

Mice were colonized with bacteria for ten days before infection. After bacterial administration, if the mice were in diestrus, they were anesthetized with ketamine (150 mg ketamine/kg) and xylazine (10 mg of xylazine/kg). Mice were intravaginally inoculated with 10^5^ PFU of wildtype HSV-2 333 and left on their backs on a heating pad for approximately 45 minutes to one hour to allow the virus to infect. Vaginal washes were collected daily for seven days post-infection, and genital pathology was monitored. Mice were euthanized when they reached a pathology of 4 or 5 (see next section).

### Genital pathology scoring

As per endpoint monitoring protocols approved by AREB, mice were monitored every day for seven days post-infection and the pathology in the genital area was recorded following a 5-point scale as previously reported (26). The scale is no infection (0), slight genital redness and inflammation (1), genital swelling and redness (2), genital and surrounding area swelling, redness and hair loss (3), genital ulceration with hair loss (4), and ulceration and hair loss in surrounding areas followed by hind limb paralysis (5). Mice were euthanized when they reached high pathology scores of 4 or 5.

### Collection of vaginal washes

30 μL of PBS was pipetted in and out of the vagina seven to eight times. A total of 50-60 μL/mouse was collected by repeating this twice. Vaginal washes were collected for up to seven days post HSV-2 infection and were stored at −80°C until required.

### Vero plaque assay

Vero cells were seeded in a 12-well plate and left for 24 h to grow to confluency in α-Minimum Essential Medium (α-MEM), supplemented with 5% fetal bovine serum (FBS), 1% penicillin-streptomycin, 1% L-glutamate and 1% HEPES. Vaginal washes were diluted from 10^-1^ to 10^-6^ in serum-free α-MEM and added to the Vero cells. Plates were incubated for 2 h and were rocked every 15 minutes to allow the virus to distribute evenly when infecting cells and to prevent the cells from drying out. After the 2 h incubation, α-MEM with FBS was added on top of the monolayers to stop new viral adsorption. The plates were placed in a 37°C incubator for 48 h. After 48 h, the media was aspirated out of each well and the cells were fixed and stained with crystal violet for approximately 15-20 minutes. The plates were then rinsed in water and left to dry overnight. Plaques were counted in the well that had 30-300 plaques under a light microscope and the plaque forming units (PFU) per mL was calculated (27).

### Immunohistochemistry of vaginal tissue

Mouse vaginal tissue was collected, placed in cassettes, and fixed in methacarn (60% methanol, 30% chloroform and 10% glacial acetic acid) for 72 h. Cassettes were transferred to 70% ethanol and samples were taken to McMaster Immunology Research Center (MIRC) Histology Core Facility for processing. Briefly, the tissue was embedded in paraffin, and slides were cut and mounted on microscope slides. Slides were then de-paraffinized and stained with a rabbit polyclonal Desmoglein-1 (DSG-1) antibody (Life Technologies, Cat #BS-6725R) as the primary antibody and a goat anti-rabbit IgG Alexa Fluor™ 488 secondary antibody (Invitrogen, Cat #A-11008) with a DAPI counterstain. An IgG isotype control (Santa Cruz Biotechnology, Cat #sc-2027) and a diluent negative control were also included. All samples were imaged on an inverted confocal laser-scanning microscope (Nikon Eclipse Ti2) at 20X magnification. For each experiment, confocal microscope settings for image acquisition and processing were identical between controls and bacteria-inoculated tissue samples. Three randomized images per sample were obtained for analysis using ImageJ software.

### Tissue processing

After inoculation with VMB species for 10 days, mice were euthanized by cervical dislocation and the vaginal tissue, spleen and iliac lymph nodes (iLN) were collected in cold Roswell Park Memorial Institute (RPMI) media (Invitrogen, Burlington, Canada). Vaginal tissue was cut open with scissors to expose the interior and then cut into smaller pieces. Collagenase A (Cedarlane, Cat #11088793001) was added to 15 mL of RPMI at a mass of 0.00157 g/mL to digest the tissue. The vaginal tissue suspension was put in 50 mL Falcon tubes and placed in a 225 RPM shaker at 37°C for one hour. The supernatant was filtered through a 40 μm filter and collected in a fresh 50 mL Falcon tube on ice. The tissue suspension was again mixed with collagenase and fresh media and digested for another hour. The suspension was then filtered through a 40 μm filter, where the remaining tissue was also pushed through the filter using the back of a syringe. All samples were centrifuged at 1500 RPM at 4°C for 10 minutes. The supernatant was discarded, and the pellet was resuspended in 200 μL of RPMI media. For spleen and iLN analysis, 40 μm filters were placed in a 6-well plate and 6 mL of RPMI media was added. The spleen or iLN was added to the filter and the back of a syringe was used to break apart the tissue. The spleen or iLN suspension was collected in a 15 mL Falcon tube and the well was washed with another 6 mL media and added to the Falcon tube. The samples were centrifuged for 5 minutes at 1500 RPMs at 4°C. The iLN pellets were resuspended in 500 μL of RPMI. Only the spleen samples were further treated with 2mL Ammonium-Chloride-Potassium lysis buffer for 4 minutes at room temperature to lyse red blood cells. Samples were washed with 10 mL PBS and placed into the centrifuge for 5 minutes at 1500 RPMs at 4°C to obtain a pellet of cells. The spleen pellets were resuspended in 6 mL of RPMI media. Cells from all samples were then counted using a hemocytometer.

### Flow cytometric analysis

Staining was done on 1x10^6^ iLN and spleen cells and all cells from the vaginal tissue due to lower yields in 200 μL volumes. Cells were stimulated with a cell stimulation cocktail of ionomycin, PMA, brefeldin A, and monensin (eBioscience™ Cell Stimulation Cocktail, Thermo Fisher Scientific, Cat #00-4975-03) overnight for 16 h as recommended by the manufacturer’s protocol. Samples were incubated with Fc block (rat anti-mouse CD16/32, BD Biosciences, Cat #553142) for 20 minutes to decrease nonspecific Fc receptor binding. Cells were then stained for cell surface markers at 4°C using the following antibodies at 1:100 dilutions for vaginal samples and 1:200 dilutions for spleen and lymph node samples: CD3 PE-CF594 (Invitrogen), CD4 PerCP Cy5.5 (Invitrogen), CD8 BV786 (Invitrogen), CD44 AF700 (BD Bioscience), CD69 PE-Cy7 (Invitrogen), and CD103 PE (Invitrogen). Cells were incubated with these antibodies for 30 minutes and then washed with PBS. Cells were then stained with the Fixable Viability Dye eFluor™ 780 (Invitrogen) at 1:1000 for 20 minutes at 4°C. To prepare for intracellular staining, cells were permeabilized and fixed using the BD Cytofix/Cytoperm™ fixation/permeabilization kit (BD Biosciences, Cat#555028) according to the manufacturer’s protocol. Cells were then stained for intracellular markers at 4°C using the following antibodies at 1:100 dilutions for vaginal samples and 1:200 dilutions for spleen and lymph node samples: IFN-γ BV421 (Invitrogen), TNF-α FITC (Invitrogen), and IL-10 BV510 (BD Biosciences). The validity of staining was verified by fluorescence minus one (FMO) control. Data were collected by flow cytometric analysis using a Cytoflex flow cytometer system (Beckman Coulter Life Sciences, Indianapolis, USA). 50 μL of cell suspensions for iLN and spleen samples and the entire 200 μL was run for vaginal tissue samples. Results were analyzed using FlowJo software (Tree Star, Ashland, USA). For the vaginal tissue, the absolute count was reported as # cells/VAG (vaginal tract) and was calculated as the direct count reported by the flow cytometer, since the entire vaginal tissue was stained and run on the flow cytometer. For the iLN and the spleen, the absolute count was reported as # cells/mL and was calculated by dividing the count from the flow cytometer by 0.05 mL to get the per mL cell count (the volume of cell suspension that was run on the machine).

### Multiplex cytokine and chemokine assay

Vaginal washes were collected from mice ten days after inoculation with bacteria. Washes were analyzed for cytokines and chemokines using the 31-Plex Mouse Cytokine/Chemokine Discovery Luminex Assay from Eve Technologies (Calgary, Canada), as per the manufacturer’s protocol.

### Statistical analysis

All statistical analysis was done using GraphPad Prism version 9.4.1 (GraphPad Software, San Diego, USA). Two-way ANOVA with Tukey’s multiple comparisons was used to determine statistical significance for quantitative plating assays and plaque assays. HSV-2 survival data was analyzed using a Log-rank (Mantel-Cox) test. A one-way ANOVA with Tukey’s multiple comparisons was used to analyze mean fluorescence intensity from histology, all flow cytometry experiments, and multiplex experiments.

## Results

### Mice inoculated every 48 h with *L. crispatus* and *G. vaginalis* maintained consistent colonization

To elucidate the cause-effect changes eubiotic and dysbiotic bacteria have on the vaginal microenvironment and susceptibility to HSV-2, a small animal model that can consistently be colonized with bacteria for a short period was required. In a previous study, we reported that *L. crispatus* and *G. vaginalis* were able to persist in the mouse vaginal tract for on average 2.6 and 1.75 days, respectively (19). Since these values are close to 48 h and 72 h, these inoculation intervals were used to determine if consistent colonization could be maintained in the model. Mice were inoculated twice 48 h or 72 h apart with the eubiotic bacteria *L. crispatus*, the dysbiotic bacteria *G. vaginalis*, or PBS as a negative control. *L. crispatus* and *G. vaginalis* loads were evaluated with quantitative plating assays, a method which we have previously validated (19).

**Figure 1:**
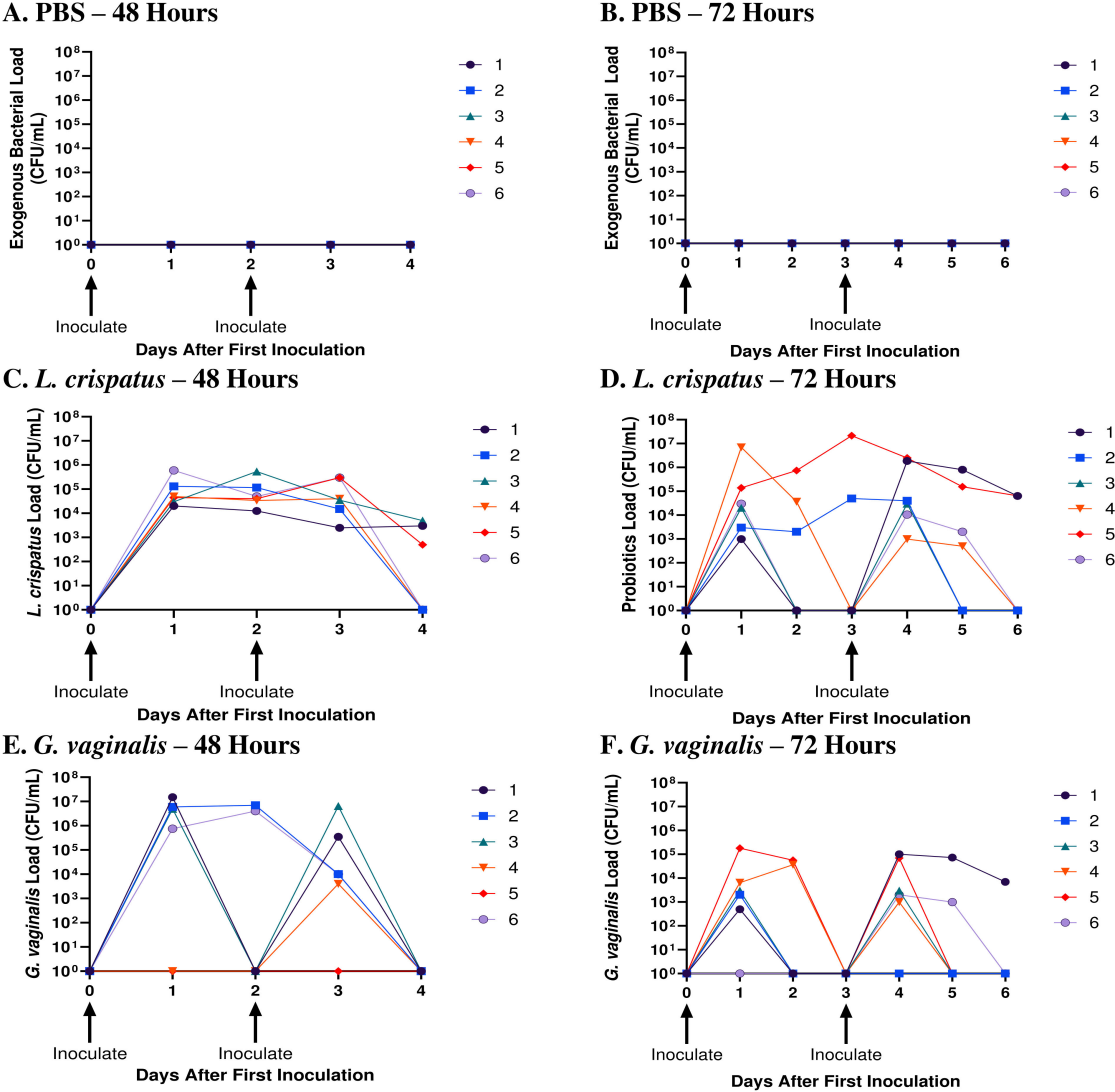
*L. crispatus* and *G. vaginalis* were consistently colonized when inoculated every 48 h. Female mice were inoculated twice 48 h or 72 h apart with 10^7^ CFU *L. crispatus*, *G. vaginalis*, or PBS as a negative control. Data are from n = 6 per group from one experiment representative of 3 independent experiments with similar results. Vaginal washes were collected post-inoculation for up to 48 h or 72 h after the second inoculation and assessed using quantitative plating assays. Bacterial colonies of the inoculated species types were counted in PBS inoculated 48 h apart **(A)**, PBS inoculated 72 h apart **(B)**, *L. crispatus* inoculated 48 h apart **(C)**, *L. crispatus* inoculated 72 h apart **(D)**, *G. vaginalis* inoculated 48 h apart **(E)**, and *G. vaginalis* inoculated 72 hours apart **(F)** groups. Different coloured points denote different mice. The data was analyzed using a two-way ANOVA with Tukey’s multiple comparisons, but no significance was found.

When mice were inoculated with PBS every 48 h (**Figure 1A**) or every 72 h (**Figure 1B**), no exogenous bacteria were detected, as expected. All mice inoculated with exogenous bacteria *L. crispatus* or *G. vaginalis* had detectable bacterial counts in the quantitative plating assays, indicating successful colonization (**Figures 1C–E**). Mice inoculated with *L. crispatus* every 48h had a high number of bacteria between the first and second inoculation, with none of the mice showing a period with low exogenous bacterial load, indicating consistent colonization (**Figure 1C**). In mice inoculated with *G. vaginalis* every 48 h, all except one showed a high exogenous bacterial count 24 h after the first inoculation (**Figure 1E**). 3/5 successfully colonized mice in this group showed declining *G. vaginalis* load at 48 h post-inoculation (**Figure 1E**). One mouse did not show any exogenous bacterial counts after the first inoculation, indicating unsuccessful colonization, which aligns with the lower rates of successful *G. vaginalis* colonization we have reported before (19). When *L. crispatus* was inoculated every 72 h, there was notably less consistency in colonization, and three mice had a period greater than 24 h with no colonization before the second inoculation (**Figure 1D**). Similarly, when mice were inoculated every 72 h with *G. vaginalis*, of the 5/6 mice colonized after the first inoculation, 3/5 mice had a period of more than 24 h with no bacteria detected (**Figure 1F**). Given the increased consistency in colonization when mice were inoculated every 48 h compared to 72 h, we proceeded with an inoculation regimen of every 48 h for this study. In this study, mice needed to be colonized with bacteria for a long enough duration of time to elicit a change in the vaginal microenvironment. Therefore, we maintained colonization for ten days (five inoculations every 48 hours). When mice were inoculated for ten days following this regimen, they consistently showed high counts of exogenous bacteria indicating short-term colonization (**Supplementary Figure S1**).

### *G. vaginalis* inoculated mice had significantly decreased survival rates, increased pathology, and high viral titers when infected with HSV-2

After determining the best inoculation regimen in the human microbiota-associated mouse models, mice were infected with HSV-2 following colonization with *L. crispatus* or *G. vaginalis* to determine if differential outcomes were seen in these mice. Clinically, a dysbiotic VMB colonized with BV-associated bacteria such as *G. vaginalis* has been shown to increase susceptibility to HSV-2 in women (13). To elucidate if this could be recapitulated in our model, mice were inoculated with *L. crispatus*, *G. vaginalis*, or PBS every 48 h for ten days (five inoculations) and on day ten, infected with a sublethal dose of wildtype HSV-2 333 at 10^5^ PFU. Administration of depot-medroxyprogesterone acetate (DMPA), a progestin-based contraceptive, before HSV-2 infection is a common practice to thin the vaginal epithelium to make mice more susceptible to HSV-2 (28), however, mice were not colonized with exogenous bacteria following DMPA administration (data not shown). Due to this, mice were staged to be in diestrus on the day of infection, the period when the epithelial barrier is thin during the normal mouse reproductive cycle (29). While not as successful as DMPA pre-treatment, this model of HSV-2 infection has previously been validated (30). The presence of bacteria following HSV-2 infection was quantitated with plating assays 24 h and five days post-infection. No bacteria were detected in the vaginal tract 24 h post-infection, indicating the virus was detrimental to vaginal bacterial growth (**Supplementary Figure S2**). At five days post-infection, no endogenous bacteria were detected, indicating the infection may be disrupting the ability for recolonization as well (**Supplementary Figure S2**).

**Figure 2:**
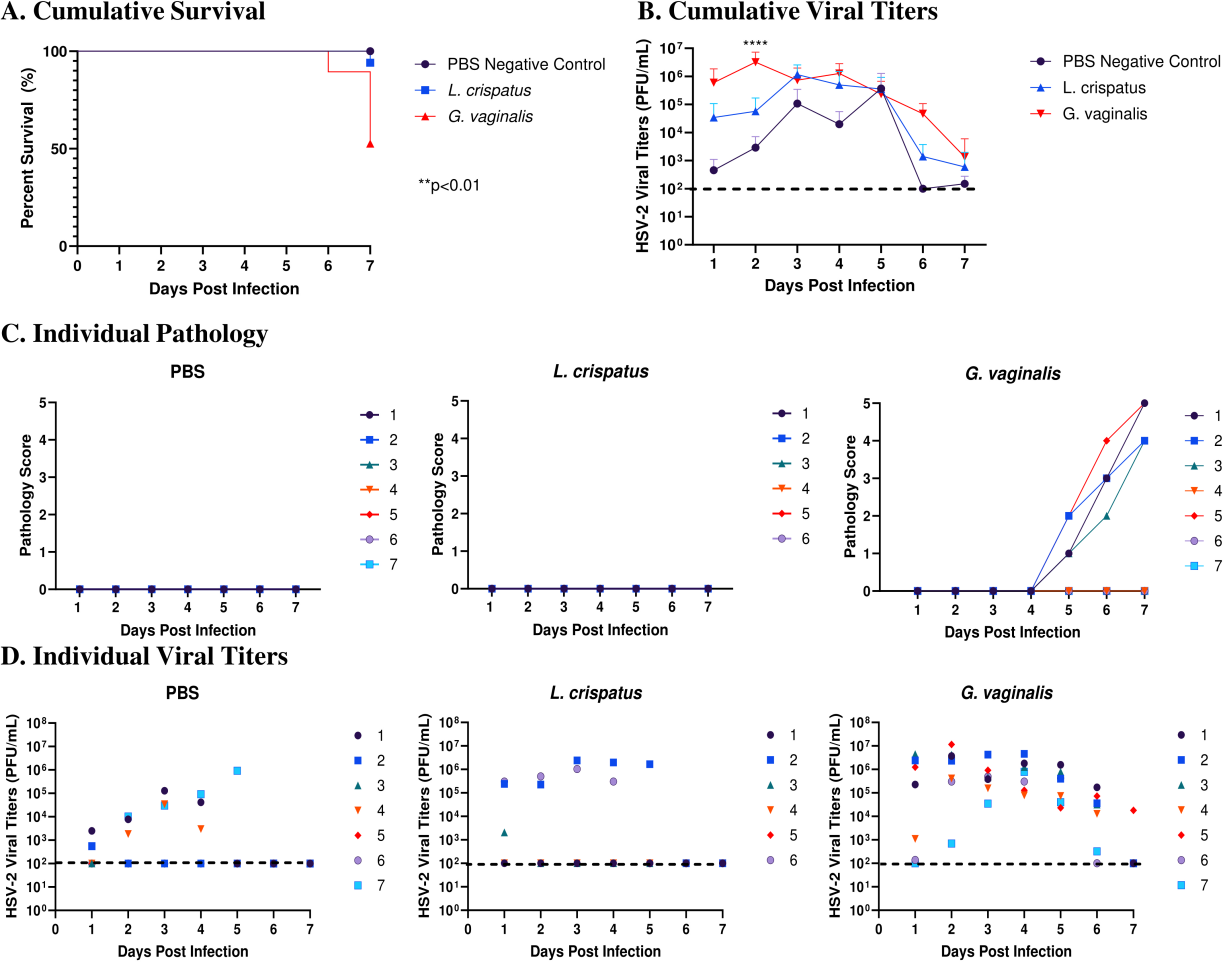
*G. vaginalis* inoculated mice showed decreased survival rates, increased pathology, and higher viral titers when infected with HSV-2 compared to *L. crispatus* and PBS-inoculated mice. Mice were administered 10^7^ CFU *L. crispatus*, *G. vaginalis*, or PBS as a no-exogenous bacteria-negative control every 48 h for 10 days. 24 h after the most recent inoculation, mice in diestrus were infected with 10^5^ PFU wildtype HSV-2 333. Data in panels **(A, B)** include cumulative data from three independent experiments (n=18 in the PBS group, n=17 in the *L. crispatus* group, and n=19 in the *G. vaginalis* group). Mice were euthanized when they reached a pathology score of 4 or 5, and survival was graphed in panel **(A)**. Vaginal washes were collected up to 7 days post-infection and viral titers were determined with a Vero plaque assay **(B)**. Vaginal pathology was recorded up to 7 days post-infection. Individual mouse data from one independent experiment representative of the three experiments was plotted for pathology **(C)** and viral titers **(D)** (n=7 per group, except *L. crispatus* n=6). Different coloured points denote different mice in panels **(C, D)**. Survival data was analyzed using a Log-rank (Mantel-Cox) test (**p<0.01). Cumulative viral titers were analyzed with a two-way ANOVA with Tukey’s multiple comparisons (****p<0.0001).

Survival and pathology were recorded, and vaginal washes were collected up to seven days post-infection to determine viral titers. Cumulative data from three independent experiments showed that *G. vaginalis* inoculated mice had significantly decreased survival rates compared to *L. crispatus* and PBS groups (**Figure 2A**). All mice survived in the PBS group, 1/17 mice did not survive in the *L. crispatus* group, and 9/19 mice succumbed to infection in the *G. vaginalis* group (**Figure 2A**). From the cumulative data, *G. vaginalis* inoculated mice also had significantly higher viral titers at two days post-infection compared to other groups (**Figure 2B**). When looking at the cumulative pathology, *G. vaginalis* inoculated mice also had increased scores compared to other groups (**Table 1**). Individual mouse data for pathology (**Figure 2C**) and viral titers (**Figure 2D**) from one independent experiment representative of the three experiments are also included. In this experiment, 4/7 mice in the *G. vaginalis* group developed severe lesions and pathology, whereas no mice in the *L. crispatus* and PBS-negative control groups developed pathology over 7 days of monitoring (**Figure 2C**). When viral titers were examined in the vaginal washes, all mice in the *G. vaginalis* group showed high viral shedding, whereas only 3/7 mice in the PBS group and 2/6 mice in the *L. crispatus* group had high viral titers (**Figure 2D**). All measures indicated *G. vaginalis* was associated with increased HSV-2 infection compared to PBS and *L. crispatus* inoculated groups. *L. crispatus* did not appear to increase or decrease susceptibility to HSV-2 *in vivo* compared to PBS controls.

**Table 1:** *G. vaginalis-*inoculated mice showed increased cumulative pathology scores compared to *L. crispatus-* and PBS-inoculated mice.

Treatment Group (Total Number of Mice)	Pathology Score	Number of Mice	Number of Days	Cumulative Pathology	Average Pathology per Mouse
PBS (n=18)	0	18	7	0	0
*L. crispatus* (n = 17)	0	16	7	0	0.2
	4	1	1	4	
*G. vaginalis* (n = 19)	0	10	7	0	2.7
	4	4	1	16	
	5	3	1	15	
	5	2	2	20	

Mice were administered 10^7^ CFU *L. crispatus*, *G. vaginalis*, or PBS every 48 h for 10 days. 24 h after the most recent inoculation, mice in diestrus were infected with 10^5^ PFU wildtype HSV-2 333. Vaginal pathology was recorded up to 7 days post-infection. Cumulative pathology is calculated by multiplying the number of mice with their maximum pathology score and the number of days that score was observed for each group. This takes into consideration that each mouse in a group can reach varying degrees of pathology through the experiment. The average pathology score per mouse was calculated by dividing the sum of cumulative pathology by the total number of mice.

### *G. vaginalis* inoculated mice had decreased Desmoglein-1 expression compared to *L. crispatus* and PBS groups

**Figure 3:**
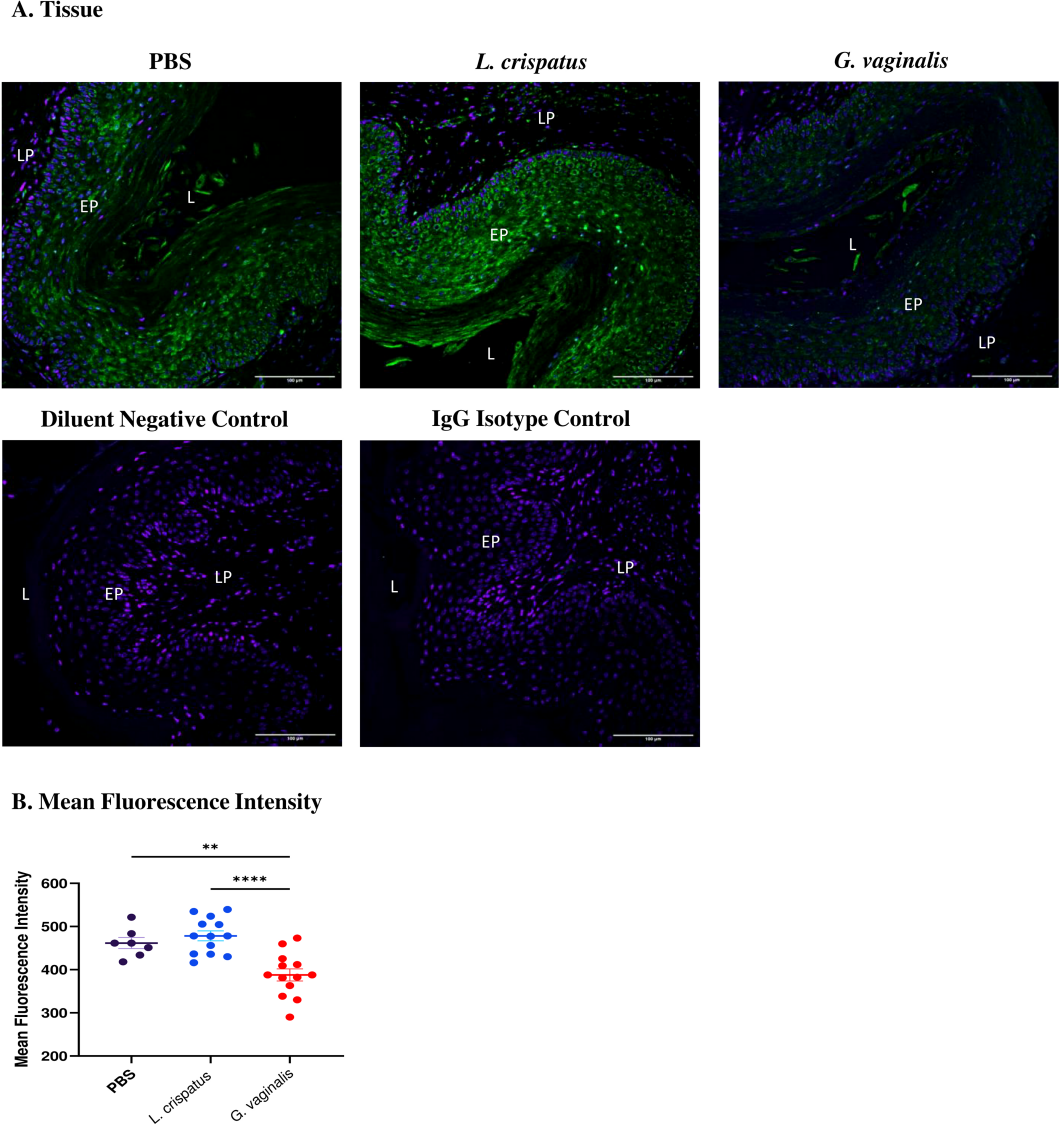
*G. vaginalis* inoculated mice had decreased Desmoglein-1 expression in the vaginal epithelium compared to *L. crispatus* and PBS groups. Mice were administered 10^7^ CFU *L. crispatus*, *G. vaginalis*, or PBS as a no-exogenous bacteria-negative control every 48 h for 10 days. Vaginal tissue was collected from the mice and fixed in methacarn. The tissue was paraffin-embedded, deparaffinized, and stained for Desmoglein-1 (green) with a DAPI counterstain (blue). Images were taken using a confocal microscope at 20X magnification **(A)**. The lumen (L), epithelium (EP), and lamina propria (LP) are indicated on all images. Mean fluorescence intensity was evaluated using three representative images per sample with ImageJ software **(B)**. Data is representative of two independent experiments (n=13 per group, except PBS n=7). The data was analyzed using a one-way ANOVA with Tukey’s multiple comparisons (**p<0.01 and ****p<0.0001).

After determining that *G. vaginalis* increased susceptibility to HSV-2 in the model, potential mechanisms as to why this is the case were explored. As the epithelium plays a vital role in susceptibility to HSV-2 (29), changes in the expression of an important epithelial barrier protein, Desmoglein-1 (DSG-1), were investigated. DSG-1 is expressed in vaginal tissue and decreased expression of this protein has previously been implicated with increased HSV-2 infection in mice (31). To determine if the VMB plays a role in DSG-1 expression, mice were inoculated every 48 h with *L. crispatus*, *G. vaginalis*, or PBS as a negative control for ten days (five inoculations). The vaginal tissue was collected on day ten and fixed and stained for DSG-1. When comparing the groups, *G. vaginalis* inoculated mice had significantly decreased expression of DSG-1 compared to *L. crispatus* and PBS groups (**Figure 3**). This was demonstrated by the decreased green fluorescent staining for DSG-1 in the *G. vaginalis* group compared to the *L. crispatus* and PBS groups (**Figure 3A**). When the mean fluorescence intensity was analyzed, there was significantly decreased expression in the *G. vaginalis* group compared to *L. crispatus* and PBS groups (**Figure 3B**). *L. crispatus* did not alter the expression of DSG-1 compared to the PBS control (**Figures 3A, B**).

### *G. vaginalis* inoculated mice had significantly decreased numbers of tissue-resident T cells compared to *L. crispatus* inoculated mice

To further elucidate why *G. vaginalis* may be increasing susceptibility to HSV-2 *in vivo*, changes in the immune microenvironment were evaluated through flow cytometry analysis. Mice were inoculated with *L. crispatus*, *G. vaginalis*, or PBS every 48 h for ten days (five inoculations). The vaginal tissue, spleen and iliac lymph nodes, which drain the reproductive tract, were collected from mice on day ten after inoculations and processed and stained for flow cytometry. The gating strategy is depicted in **Supplementary Figure S3**. No significant differences were seen in the cells from the spleen or the lymph nodes, indicating that the effect of vaginal bacterial colonization was site-specific and limited to local tissues (**Supplementary Figure S4**).

**Figure 4:**
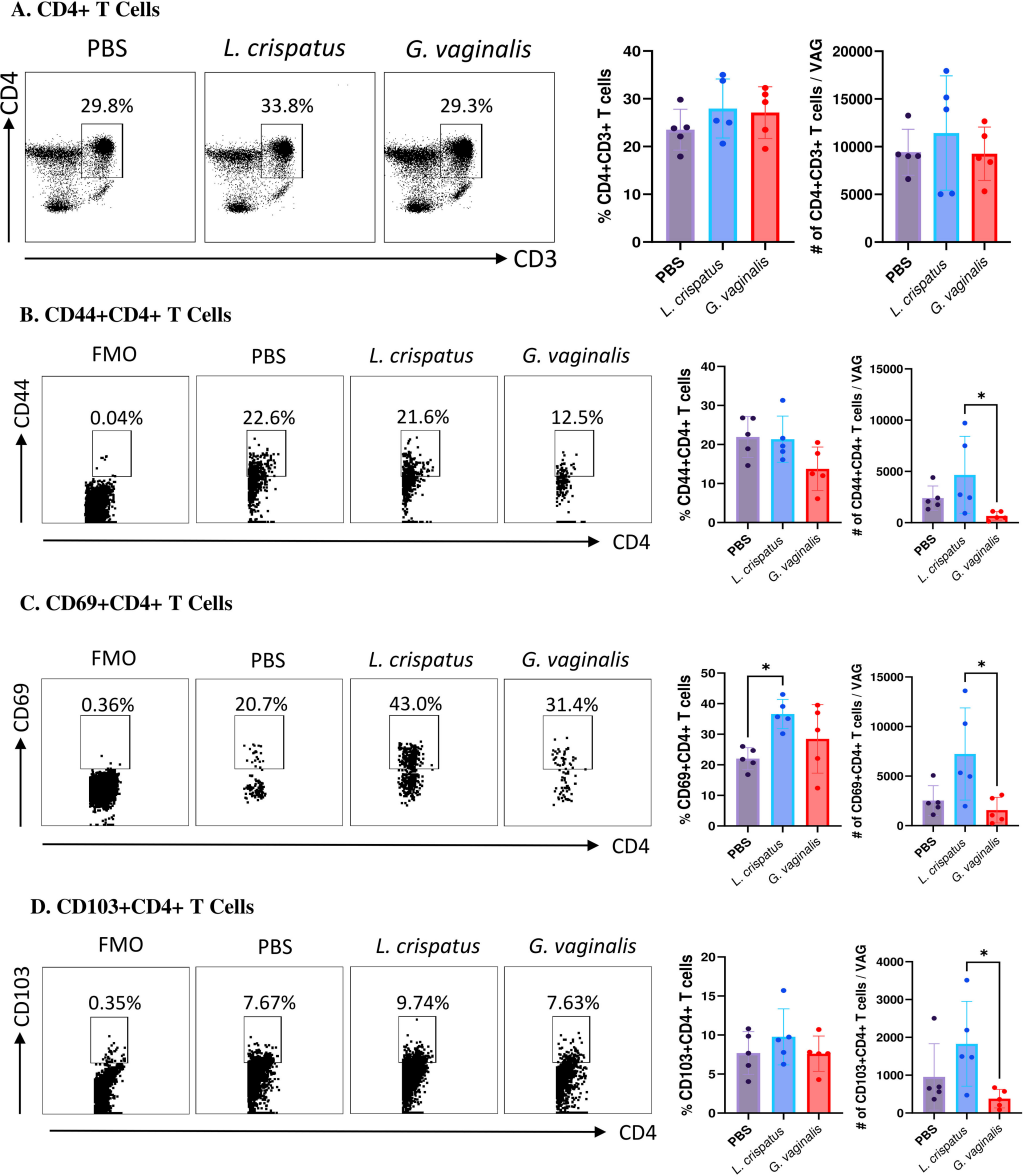
*G. vaginalis* inoculated mice had decreased activated and mucosal CD4+ T cells in the vaginal tract compared to L. crispatus inoculated mice. Female mice were intravaginally inoculated with 10^7^ CFU *L. crispatus*, *G. vaginalis*, or PBS as a no-exogenous bacteria-negative control every 48 h for 10 days. On day 10 of the experiment, the vaginal tissue (VAG) was collected, processed, and stimulated for 16 h. Cells were stained for Live/Dead staining, CD3, CD4, CD44, CD69, and CD103 and ran on the Cytoflex flow cytometer and analyzed using FloJo software. The percent population and absolute count of CD4+ T cells are shown in panel **(A)**. The percent population and absolute number of CD44+CD4+ T cells **(B)**, CD69+CD4+ T cells **(C)**, and CD103+CD4+ T cells **(D)** are depicted as well. Data are from n=5 per group, from one experiment representing three independent experiments. Data was analysed using a one-way ANOVA with Tukey’s multiple comparisons (*p<0.05).

Since T cells have been shown to play a critical role in protection against HSV-2, our analysis was focused on these cells (32, 33). Vaginal cells were first gated on CD4+CD3+ T cells. Inoculation with *L. crispatus* or *G. vaginalis* did not significantly alter the proportions or absolute number of CD4+ T cells (**Figure 4A**), indicating these bacteria did not affect the overall numbers of CD4+ T cells in the vaginal mucosa. Next, the quality of T cell response in the vaginal tract following inoculation with different bacteria was evaluated by examining CD4+ T cells expressing the tissue-resident marker CD103 (34), the tissue-resident and activation marker CD69 (35), and the tissue-resident and adhesion marker CD44 (36). *G. vaginalis* inoculated mice had significantly decreased absolute numbers of CD44+CD4+ T cells (**Figure 4B**), CD69+CD4+ T cells (**Figure 4C**), and CD103+CD4+ T cells (**Figure 4D**) compared to *L. crispatus* inoculated mice. *L. crispatus* inoculated mice had significantly increased proportions of CD69+CD4+ T cells compared to the PBS group. All of these markers are mucosal tissue residency markers, signifying there was a decrease in tissue-resident helper T cells in the vaginal immune environment in the *G. vaginalis* inoculated mice compared to *L. crispatus*. Decreased numbers of resident CD4+ T cells in the vaginal tissue could be linked to the increased susceptibility to HSV-2 in the *G. vaginalis* inoculated mice.

**Figure 5:**
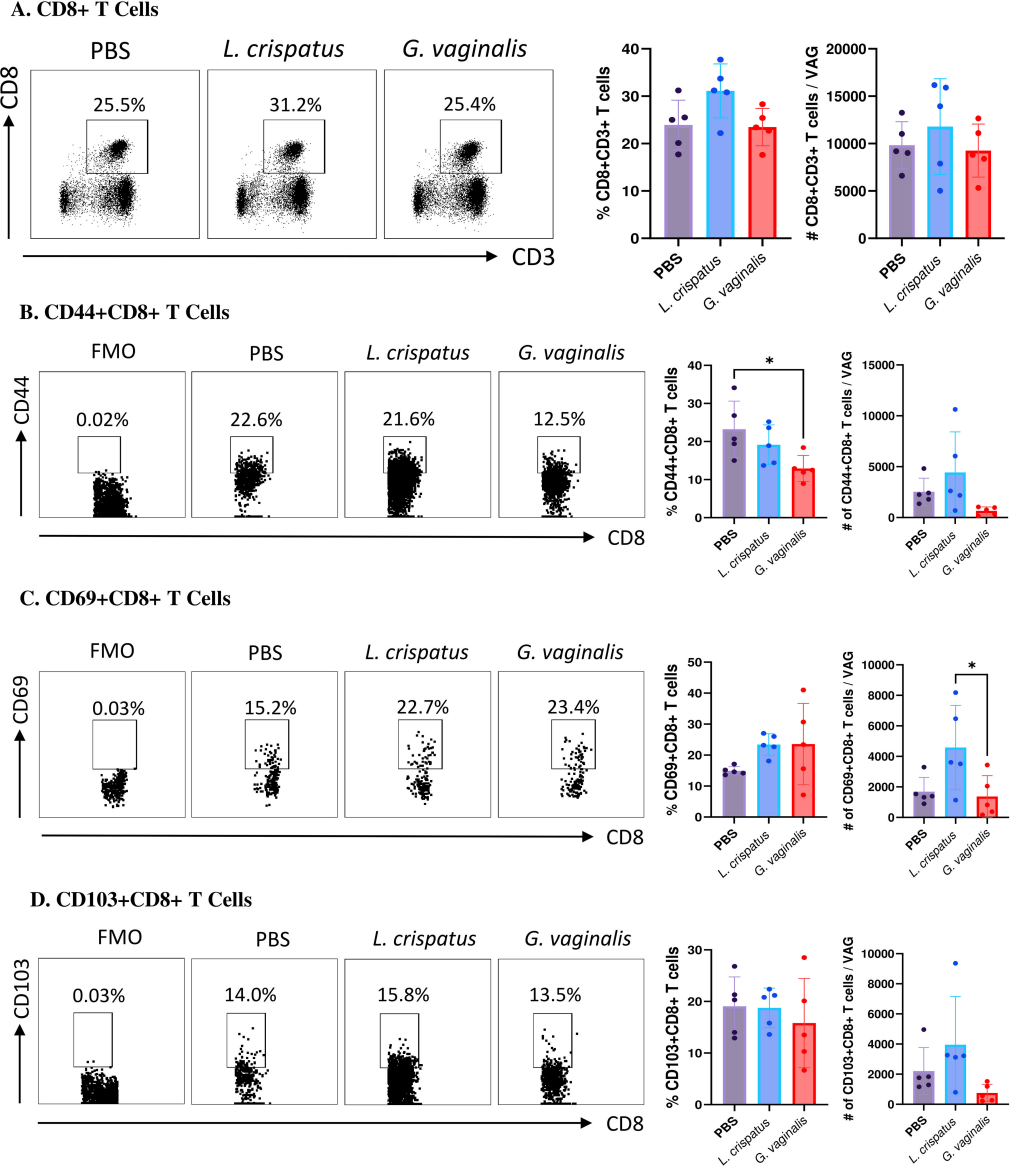
*G. vaginalis* inoculated mice had decreased activated and mucosal CD8+ T cells in the vaginal tract compared to *L. crispatus* inoculated mice. Female mice were intravaginally inoculated with 10^7^ CFU *L. crispatus*, *G. vaginalis*, or PBS as a no-exogenous bacteria-negative control every 48 h for 10 days. On day 10 of the experiment, the vaginal tissue (VAG) was collected, processed, and stimulated for 16 h. Cells were stained for Live/Dead staining, CD3, CD8, CD44, CD69, and CD103, and ran on the Cytoflex flow cytometer and analyzed using FloJo software. The percent population and absolute number of CD8+ T cells are shown in panel **(A)**. The percent population and absolute number of CD44+CD8+ T cells **(B)**, CD69+CD8+ T cells **(C)**, and CD103+CD8+ T cells **(D)** are depicted as well. Data are from n=5 per group, from one experiment representing three independent experiments. Data was analyzed using a one-way ANOVA with Tukey’s multiple comparisons (*p<0.05).

Next, the CD8+CD3+ T cell compartment was examined (**Figure 5A**). Similar to the CD4+ T cells, there was not a significant change in the CD8+ T cells in the *G. vaginalis* or *L. crispatus* inoculated mice compared to the PBS group, indicating the bacterial inoculations did not significantly alter the total amount of CD8+ T cells in the vaginal mucosa. Once again, cells were further gated for mucosal markers including CD44+CD8+ T cells, CD69+CD8+ T cells, and CD103+CD8+ T cells. When compared to the PBS group, *G. vaginalis* inoculated mice had significantly decreased percentages and trends towards decreased absolute numbers of CD44+CD8+ T cells (**Figure 5B**). There was also significantly decreased absolute counts of CD69+CD8+ T cells (**Figure 5C**). There were trends toward decreased absolute counts of CD103+CD8+ T cells in the *G. vaginalis* group (**Figure 5D**). Although not as strong as the data for CD4+ T cells, there was a decrease in mucosal CD8+ T cells in *G. vaginalis* inoculated mice. This could also contribute to the increased susceptibility to HSV-2 in the *G. vaginalis* group.

### *G. vaginalis* inoculated mice had significantly increased levels of T cells expressing TNF-α *and IFN-*γ while *L. crispatus* inoculation enhanced IL-10 expression in T cells

In conjunction with mucosal cell surface markers, cytokine production by T cells was also assessed using flow cytometry to determine functional T cell populations in VMB-administered mice. Mice were inoculated with *L. crispatus*, *G. vaginalis*, or PBS as a negative control for ten days (five inoculations every 48 h). Following bacterial inoculation, the vaginal tissue, spleen and iliac lymph nodes were collected from mice on day ten after inoculations and processed and stained for flow cytometry. Cells were gated for CD4+ and CD8+ T cells and then further gated for the proinflammatory cytokines TNF-α and IFN-γ and the regulatory cytokine IL-10. Once again, no significant differences were noted in the cytokine expression by T cells from the spleen or the lymph nodes (**Supplementary Figure S5**), indicating that the effect of bacterial inoculation was site-specific and limited to the vaginal tract.

**Figure 6:**
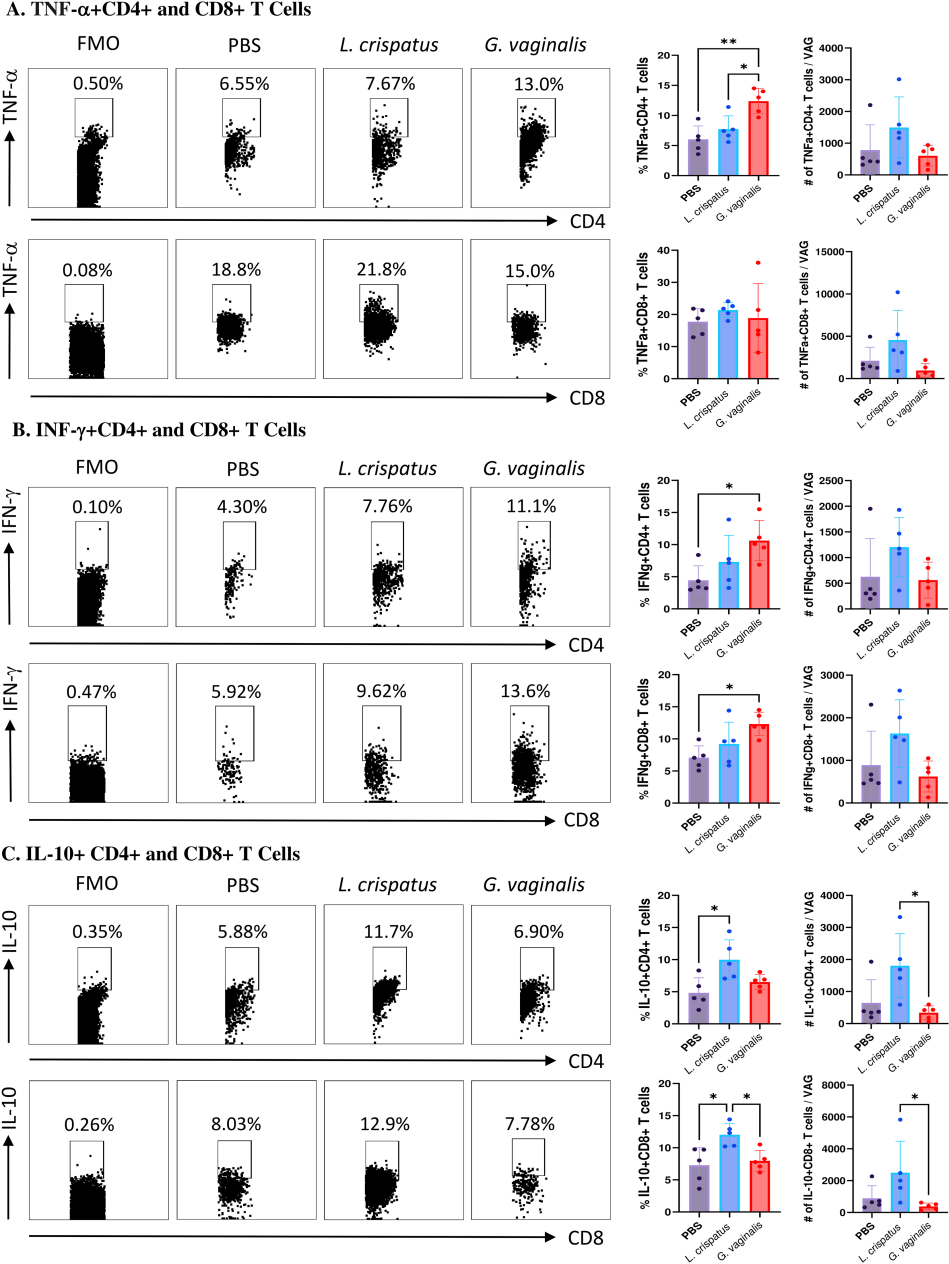
*G. vaginalis* inoculated mice had increased T cells expressing inflammatory cytokines and *L. crispatus* treated mice had increased T cells expressing regulatory cytokines in the vaginal tract. Female mice were intravaginally inoculated with 10^7^ CFU *L. crispatus*, *G. vaginalis*, or PBS as a no-exogenous bacteria-negative control every 48 h for 10 days. On day 10 of the experiment, the vaginal tissue (VAG) was collected, processed, and stimulated for 16 h. Cells were stained for Live/Dead staining, CD3, CD4, CD8, TNF-α, IFN-γ, and IL-10, and ran on the Cytoflex flow cytometer and analyzed using FloJo software. CD4+ and CD8+ T cells expressing TNF-α **(A)**, IFN-γ **(B)**, and IL-10 **(C)** are depicted. Data are from n=5 per group, from one experiment representing three independent experiments. Data was analyzed using a one-way ANOVA with Tukey’s multiple comparisons (*p<0.05 and **p<0.01).

*G. vaginalis* inoculated mice had significantly increased proportions of TNF-α+CD4+ T cells compared to PBS and *L. crispatus*; however, the absolute numbers were not significantly different, indicating a possible selective enrichment of TNF-α producing CD4+ T cells (**Figure 6A**). There was no significant difference in the TNF-α+CD8+ T cells (**Figure 6A**). *G. vaginalis* inoculated mice had significantly increased proportions of IFN-γ+CD4+ and IFN-γ+CD8+ T cells compared to the PBS group (**Figure 6B**), but no difference in the absolute counts, once again indicating the possibility of selective enrichment. Lastly, the *L. crispatus* inoculated group has significantly increased proportions of IL-10+CD4+ T cells compared to PBS and significantly increased absolute counts of IL-10+CD4+ T cells compared to *G. vaginalis* (**Figure 6C**). *L. crispatus* inoculated mice also had significantly increased proportions of IL-10+CD8+ T cells compared to PBS and *G. vaginalis* groups and significantly increased absolute counts compared to *G. vaginalis* (**Figure 6C**). Overall, these results suggest *G. vaginalis* inoculation was associated with an inflammatory signature in vaginal T cells while *L. crispatus* inoculation was associated with regulatory cytokine production by vaginal T cells.

### *G. vaginalis* inoculated mice had increased inflammatory cytokine and chemokine production compared to *L. crispatus*

**Figure 7:**
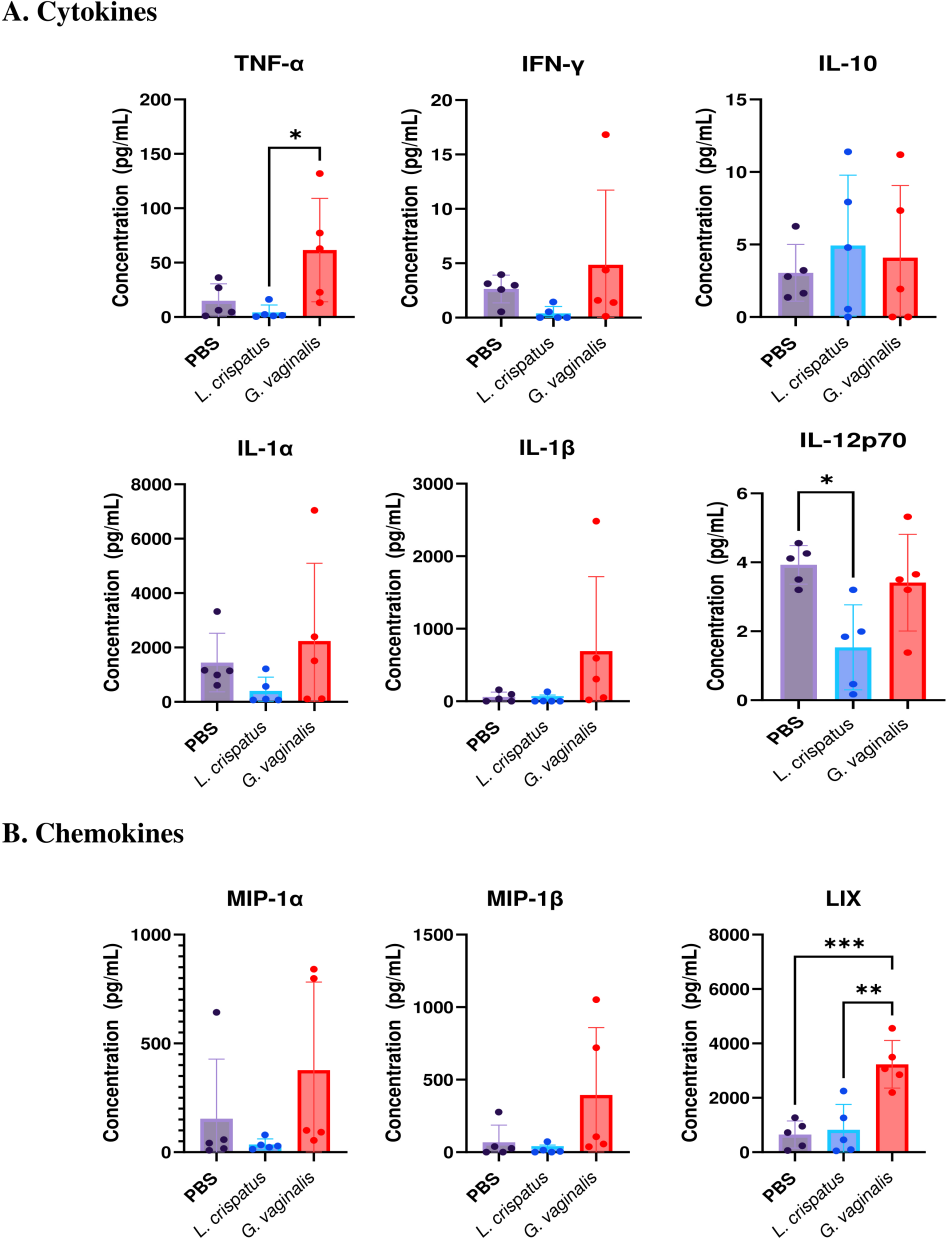
*G. vaginalis* inoculated mice had increased inflammatory cytokine and chemokine protein levels in the vaginal tract. Female mice were intravaginally inoculated with 10^7^ CFU *L. crispatus*, *G. vaginalis*, or PBS as a no-exogenous bacteria-negative control every 48 h for 10 days. On day 10 of the experiment, vaginal washes were collected and sent to Eve Technologies to perform a 31-Plex mouse cytokine/chemokine assay. All significant cytokine **(A)** and chemokine **(B)** data are shown above. Data are from n=5 per group, from one independent experiment. Data was analyzed using a one-way ANOVA with Tukey’s multiple comparisons (*p<0.05, **p<0.01, and ***p<0.001).

Since the flow cytometric analysis indicated functional differences in T cell populations, we next examined if there were changes in the cytokine levels in the vaginal immune milieu following colonization with different bacteria. A multiplex assay was used to assess cytokine and chemokine levels after bacterial inoculation. Mice were inoculated for ten days (five inoculations 48 h apart) with *L. crispatus*, *G. vaginalis*, or PBS as a negative control. On day ten, vaginal washes were collected from mice and a 31-Plex mouse cytokine/chemokine assay was conducted to analyze cytokine levels in the vaginal tract. While the majority of the 31 cytokines and chemokines examined did not show any significant changes following colonization with different bacteria, some key cytokines and chemokines were altered. Overall *G. vaginalis* was associated with an inflammatory signature in the vaginal canal compared to *L. crispatus*. In congruence with the flow cytometry experiments, *G. vaginalis* inoculated mice had significantly increased TNF-α levels in the vaginal secretions compared to *L. crispatus* (**Figure 7A**). There were also trends towards increased IFN-γ, IL-1α, and IL-1β levels in the *G. vaginalis* group. *L. crispatus* had significantly decreased levels of IL-12p70 expression compared to PBS (**Figure 7A**). IL-10 levels were not significantly affected in bacteria-inoculated mice (**Figure 7A**). When the expression of chemokines was examined, *G. vaginalis* also had increased expression of chemokines that attract inflammatory cells, including a significant increase in LIX, a chemokine known to attract neutrophils (37), and a trend towards increased expression of MIP-1α and MIP-1β (**Figure 7B**). Other cytokines and chemokines from the 31-Plex assay did not show any trends or significance (data not shown).

## Discussion

To prevent the spread of STIs in vulnerable populations, it is important to understand why women are disproportionately susceptible to viral STIs such as HSV-2. The distinct VMB of the female reproductive tract is thought to play a role in this susceptibility bias, which is supported by clinical and epidemiological evidence (12–14). However, epidemiological and *in vitro* studies are limited in their ability to elucidate cause-effect mechanisms of how VMB composition affects HSV-2 susceptibility. *In vitro* studies do not fully mimic a physiological system, and epidemiological studies are usually retrospective and typically demonstrate correlation rather than cause-effect relationships. Using human microbiota-associated (HMA) mouse models that harbour human VMB species can address some of the shortcomings of other studies and provide a physiologically relevant model that can be experimentally manipulated. There have been attempts to colonize mice with *Lactobacillus* species and *G. vaginalis* to assess physiological outcomes in the vaginal microenvironment, however, these models have not examined the outcomes of common sexually transmitted viruses, like HSV-2, in the presence of distinct bacteria present in the human VMB (20, 38–44). There is clinical evidence to suggest a dysbiotic VMB is associated with increased susceptibility to HSV-2 in women (12–14), however, biological mechanisms are lacking in the literature. In this study, we used our novel HMA models that were previously reported (19) to elucidate mechanisms as to how the VMB may affect susceptibility to HSV-2.

We first tested our models under HSV-2 infection to determine if inoculation with the dysbiotic bacteria *G. vaginalis* would mimic clinical evidence and increase susceptibility to HSV-2 *in vivo.* In the VMB associated mouse models, there was decreased survival, increased pathology, and higher viral titers in mice inoculated with *G. vaginalis* compared to *L. crispatus* and PBS mice. Normal mice that are at different stages of the reproductive cycle are typically not susceptible to HSV-2 unless they are experimentally manipulated to enable thinning of the vaginal epithelium (29, 30). We have previously reported when normal mice are infected with HSV-2 at a dose of 10^5^ PFU, mice do not show pathology and survive, similar to the results from PBS mice in this study (29). No significant increase in susceptibility was seen for *L. crispatus* inoculated mice. However, we did observe significantly enhanced susceptibility in the *G. vaginalis* inoculated group compared to the PBS and *L. crispatus* groups. We have previously reported that the mouse VMB has a finite niche, and colonization with exogenous bacteria displaces the endogenous mouse microbiota (19). Due to the low levels of endogenous species, we posit that the change in susceptibility was due to the exogenous VMB species we inoculated. Importantly, no live bacteria could be detected in the vaginal tract following HSV-2 infection, indicating that any differences in susceptibility were due to changes that occurred in the vaginal microenvironment before exposure to the virus. The mice which developed pathology and did not survive followed the typical course of *in vivo* infection which has previously been described in several studies and published extensively (26, 28, 30, 31, 45, 46). Similar to other published studies, high viral titers were detected in the vaginal tract for 3-5 days post-infection at a time when the virus is known to replicate in the vaginal epithelium (30). During this time, the innate and early adaptive antiviral immune responses have been shown to be activated in order to contain the viral replication (47, 48). At the sublethal dose of challenge, in the *L. crispatus* and PBS groups, we would posit that the immune system was able to control the spread of the virus throughout the epithelium, which is why the viral titers began to decrease at around 4-5 days post-infection. In the *G. vaginalis* group, however, some mice rapidly developed pathology after five days of infection; we would predict that the virus had evaded the immune system, successfully obliterated the epithelium and spread to the paracervical ganglia and the central nervous system (28). By day 6-7 of infection, the decrease in vaginal viral titers even in mice that developed pathology is likely because the virus is no longer replicating in the vaginal epithelium and has entered the nervous system (30). Taken together, our results show that *G. vaginalis* inoculation shaped the vaginal microenvironment to be conducive to successful infection with the sublethal dose of HSV-2 prior to infection, ultimately leading to the pathology and lower survival rates in this group.

While this is the first study to report the role of the VMB on HSV-2 infection *in vivo*, other groups have also reported inoculation of *G. vaginalis* or other dysbiotic species to be associated with negative health outcomes. *G. vaginalis* has been implicated in adverse pregnancy outcomes in multiple models. Studies have shown that rabbits administered *G. vaginalis* to the upper reproductive tract have significantly lower live birth rates, lower fetal weights, and neural pathology in the fetus (49, 50). Mice inoculated intravaginally with *G. vaginalis* have been shown to have increased inflammation, soluble E-cadherin, and cervical remodelling at earlier time points of gestation. While cervical remodelling is a normal part of parturition, earlier indications can be implicated with pre-term birth (39). Studies have also connected dysbiotic bacteria and urinary tract infections. One *in vivo* study found bladder exposure to *G. vaginalis* triggered *Escherichia coli* egress from latent bladder reservoirs. *G. vaginalis* exposure was also sufficient to cause epithelial apoptosis in the bladder and to induce kidney injury via an IL-1 receptor pathway (51). Perhaps most relevant to this study, there are several mouse studies implicating a dysbiotic vaginal microbiota with an increased risk of bacterial STIs and HIV. One study found that infection with Group B *Streptococcal* species decreased the stability of the mouse VMB, and endogenous *Staphylococcal* abundance in the mouse VMB correlated with increased infection outcomes (52). Another study found that humanized mice administered MPA had increased VMB diversity, which resulted in enhanced HIV susceptibility (23). It was also found that the endogenous VMB of BALB/c mice consists mainly of *Pseudomonas* and *Janthinobacterium*, and that infection with HSV-2 shifted the vaginal microbiota of these mice to contain more dysbiotic species *Staphylococcus* and *Escherichia* (53). While these studies all implicate *G. vaginalis* and other dysbiotic bacteria with negative health outcomes, there are very few studies in the literature reporting the use of *in vivo* models to elucidate the underlying mechanisms to explain how the vaginal bacteria may affect susceptibility to infections. To our knowledge, this is the first study that administered eubiotic and dysbiotic human VMB species to mice to elucidate the effect these bacteria have on HSV-2 infection outcomes *in vivo*.

As previously mentioned, the main purpose of HMA models is to determine cause-effect mechanisms of how the VMB may affect susceptibility to infections such as HSV-2. In this study, we first determined the role of the VMB on barrier integrity, as the vaginal epithelial barrier is essential to HSV-2 infection (28, 54). Previous studies have shown that the vaginal epithelium plays a critical role in enabling HSV-2 infection since thin and/or disrupted epithelium allows the virus to rapidly gain entry into the lamina propria of the mucosa and establish infection (31, 55). Given the nature of how HSV-2 infects, we wanted to examine if human VMB species have an effect on the vaginal epithelium, which can help explain why *G. vaginalis* was associated with increased susceptibility to HSV-2 in our mice. We found *G. vaginalis* inoculation resulted in decreased Desmoglein-1 staining, which is a desmosomal adhesion protein found in the vaginal epithelium of humans and mice (22, 56). Desmosomes are a major intracellular adhesion junction at the basolateral membrane (57). The reduction of this protein in the *G. vaginalis* group provides a potential mechanism for the HSV-2 virus to breach the epithelium and infect the central nervous system, leading to increased HSV-2 pathology in this group. Another study has also found inoculation of mice with *G. vaginalis* decreases the expression of junctional proteins such as DSG-1 expression in the vaginal tract of BALB/c mice (58). *G. vaginalis* has also been shown in other clinical studies to disrupt the barrier by degrading mucin through the production of sialidase (59) and promoting epithelial cell shedding (60). Overall, our results align with previously published data on *G. vaginalis* and epithelial barrier disruption and provide a mechanism for how this dysbiotic bacteria may increase HSV-2 infection outcomes.

Another important factor in HSV-2 infection is the immune response to the virus. The composition of the VMB is known to alter immune responses in the vaginal mucosa, which can affect susceptibility to HSV-2 (61, 62). In our study, we found *G. vaginalis* was associated with decreased overall numbers of tissue-resident T cells. We saw a decrease in the numbers of T cells expressing CD69, CD103, and CD44 in *G. vaginalis* mice compared to *L. crispatus* inoculated mice. The presence of these markers can be used to identify activated mature mucosal immune cells that are available locally in the mucosal tissue to fight infection (34–36). *G. vaginalis* inoculated mice had significantly decreased numbers of activated CD69+ T cells, and T cells expressing mucosal homing markers CD103 and CD44 compared to *L. crispatus* mice in both the CD4 and CD8 compartments. Since the overall number of total CD4+ and CD8+ T cells was not changed in the vaginal tract of these mice, this could indicate a selective reduction in mature T cells that can readily fight HSV-2 upon infection. Whilst out of the scope of this study, other groups have investigated how *G. vaginalis* may alter T cell responses. In one study, *G. vaginalis* was found to possess slight immune stimulating activity against T cells. At high doses, *G. vaginalis* significantly increased proliferation and cytokine secretion in lymphocytes (63). This leads to a defective inflammatory response and atypical low-grade inflammation. Importantly, this atypical cytokine production also plays an important role in T cell activation and chemotaxis, which could play a role in the reduced activation markers seen in *G. vaginalis* inoculated mice (64).

We also found *G. vaginalis* inoculated mice to be associated with increased inflammatory cytokine and chemokine production. In HSV-2 infection, an influx of immune cells is a part of a productive immune response (65). However, having a state of constant non-specific inflammation can be associated with an increased risk of sexually transmitted infections such as HIV (66). Chronic inflammation can also damage healthy cells and tissues (67). Pro-inflammatory cytokines have also been attributed to a disruption of the epithelial barrier (68). *G. vaginalis* has been implicated with inflammation in multiple clinical and *in vitro* studies (69–74). Some *in vivo* studies have also indicated this. In one study, *G. vaginalis* has been shown to increase transcript levels of cytokines IL-8, IL-1β, and IL-10 in the cervices of mice and protein levels of IL-6 in the cervicovaginal fluid (39). Another group has also reported increased protein levels of pro-inflammatory cytokines TNF-α, IL-1β, and IL-6 in the vaginal tissue of *G. vaginalis* inoculated mice compared to control mice (40–42). In addition, the authors reported a higher number of inflammatory markers myeloperoxidase, iNOS, and COX-2 levels in the vaginal tissues of *G. vaginalis* inoculated mice. Additionally, when histology was performed on vaginal tissue, there appeared to be substantial edema and immune cell infiltration into the superficial mucosal layers (75). In our study, we also saw an increase in classic inflammatory cytokines TNF-α, IL-1α and IL-1β in *G. vaginalis* inoculated mice, similar to what has previously been reported. We also saw increased amounts of MIP-1α, MIP-1β, and LIX, which are potent recruiters of immune cells such as neutrophils (37). In our study, we also saw an increase of IL-10+ T cells in *L. crispatus* inoculated mice, but not *G. vaginalis*. IL-10 is a regulatory cytokine that plays a role in preventing damage to the host and maintaining normal tissue homeostasis (76), so, consistently, the eubiotic bacteria *L. crispatus* had increased IL-10+ T cells. In the multiplex assay, IL-10 protein levels were however unaffected by bacteria inoculation. The multiplex assay used vaginal washes, and all of the flow cytometry data was from whole tissue homogenates, so these differences may be due to the difference in sample types. We also saw a significant decrease in pro-inflammatory cytokine IL-12p70 protein levels in *L. crispatus* inoculated mice, further indicating a potential regulatory role for *L. crispatus in vivo*.

In summary, this study provides new and important insights into the mechanism behind increased HSV-2 infection due to a dysbiotic VMB using an *in vivo* model. While clinical studies have provided significant insights regarding the correlation between colonization by dysbiotic species such as *G. vaginalis* with increased susceptibility to HSV-2, mechanistic studies examining cause-effect relationships have not been possible due to the lack of animal models. In this study, we identified the disruption of the vaginal epithelial barrier and the reduction of protective immune responses in *G. vaginalis* inoculated mice, which likely contributed to the increased HSV-2 infection outcomes seen in these mice. The current model can be translated to humanized mice as well as germ-free mice to study the role of the VMB on HIV susceptibility and to further understand the immune development associated with the VMB. Moving forward, mouse models that harbour human vaginal microbiota species can play a critical role in better understanding the roles of the VMB in female reproductive health.

## Data Availability

The raw data supporting the conclusions of this article will be made available by the authors, without undue reservation.

## Supplementary material

Supplementary Figure 1 Mice in estrus were able to stay colonized with exogenous bacteria. Female mice were inoculated five times every 48 h with 10^7^ CFU *L. crispatus* or *G. vaginalis.* Data are from n = 4 per group from one independent experiment. Vaginal washes were collected every day until 24 h after the fifth inoculation (day 9) and assessed using quantitative plating assays. Bacterial colonies of the inoculated species types were counted in *L. crispatus* inoculated **(A)** and *G. vaginalis* inoculated **(B)** mice. Different coloured points denote different mice. Individual mouse data is plotted beside the cumulative graphs. Red dots in these graphs denote the mouse was in estrus and black dots denote the mouse was in diestrus on that day. The arrows indicate the day mice were inoculated with bacteria. The data was analyzed using a two-way ANOVA with Tukey’s multiple comparisons, but no significance was found.

Supplementary Figure 2 No bacteria was detected in the vaginal tract following HSV-2 infection. Female mice were inoculated five times every 48 h with 10^7^ CFU *L. crispatus, G. vaginalis*, or PBS as a no-exogenous bacteria negative control. Vaginal washes were collected 24 h before intravaginal infection with 10^5^ PFU wildtype HSV-2, as well as one- and five-days post infection. Bacterial load was assessed using quantitative plating assays. Bacterial colonies were counted in n=14 mice and plotted. Different dots denote different mice. The data was analyzed with a one-way ANOVA with Tukey’s multiple comparisons (*p<0.05).

Supplementary Figure 3 Gating Strategy for Flow Cytometry. Female mice were intravaginally inoculated with 10^7^ CFU *L. crispatus*, *G. vaginalis*, or PBS as a no-exogenous bacteria-negative control every 48 h for 10 days. On day 10 of the experiment, the vaginal tissue was collected, processed, and stimulated for 16 h. Cells were stained for Live/Dead staining, CD3, CD4, and CD8 (other markers for tissue-resident and functional T cells were also included). The gating strategy up to CD4+CD3+ and CD8+CD3+ T cells is depicted above.

Supplementary Figure 4 There were no differences in T cell populations in the spleen and lymph nodes of VMB-inoculated mice. Female mice were intravaginally inoculated with 10^7^ CFU, *L. crispatus*, *G. vaginalis*, or PBS as a no-exogenous bacteria-negative control every 48 h for 10 days. On day 10 of the experiment, the spleen and iliac lymph nodes (LN) were collected, processed, and stimulated for 16 h. Cells were stained for Live/Dead staining, CD3, CD4, CD8, CD44, CD103, and CD69, ran on the Cytoflex flow cytometer, and analyzed using FloJo software. The gating strategy is shown in panel **(A)**. The percent population and absolute count of CD44+CD4+ T cells in the spleen **(B)** and LN **(C)**, CD44+CD8+ T cells in the spleen **(D)** and LN **(E)**, CD69+CD4+ T cells in the spleen **(F)** and LN **(G)**, CD69+CD8+ T cells in the spleen **(H)** and LN **(I)**, CD103+CD4+ T cells in the spleen **(J)** and LN **(K)**, and CD103+CD8+ T cells in the spleen **(L)** and LN **(M)**, are depicted above. Data are from n=7 per group, from one experiment representing three independent experiments. Data was analyzed using a one-way ANOVA with Tukey’s multiple comparisons, but no significance was found.

Supplementary Figure 5 There were no differences in cytokine-producing T cells in the spleen and lymph nodes of VMB-inoculated mice. Female mice were intravaginally inoculated with 10^7^ CFU *L. crispatus*, *G. vaginalis*, or PBS as a no-exogenous bacteria-negative control every 48 h for 10 days. On day 10 of the experiment, the spleen and iliac lymph nodes (LN) were collected, processed, and stimulated for 16 h. Cells were stained for Live/Dead staining, CD3, CD4, CD8, TNF-α, IFN-γ, and IL-10, ran on the Cytoflex flow cytometer, and analyzed using FloJo software. The gating strategy is shown in panel **(A)**. The percent population and absolute count of TNF-α+CD4+ T cells in the spleen **(B)** and LN **(C)**, TNF-α+CD8+ T cells in the spleen **(D)** and LN **(E)**, IFN-γ+CD4+ T cells in the spleen **(F)** and LN **(G)**, IFN-γ+CD8+ T cells in the spleen **(H)** and LN **(I)**, IL-10+CD4+ T cells in the spleen **(J)** and LN **(K)**, and IL-10+CD8+ T cells in the spleen **(L)** and LN **(M)**, are depicted above. Data are from n=7 per group, from one experiment representing three independent experiments. Data was analyzed using a one-way ANOVA with Tukey’s multiple comparisons, but no significance was found.
