# Supplementary figures and images for: G. vaginalis increases HSV-2 infection by decreasing vaginal barrier integrity and increasing inflammation in vivo
Source: Front Immunol. 2024 Nov 22;15:1487726. doi: 10.3389/fimmu.2024.1487726 (PMC11621107; doi:10.3389/fimmu.2024.1487726)

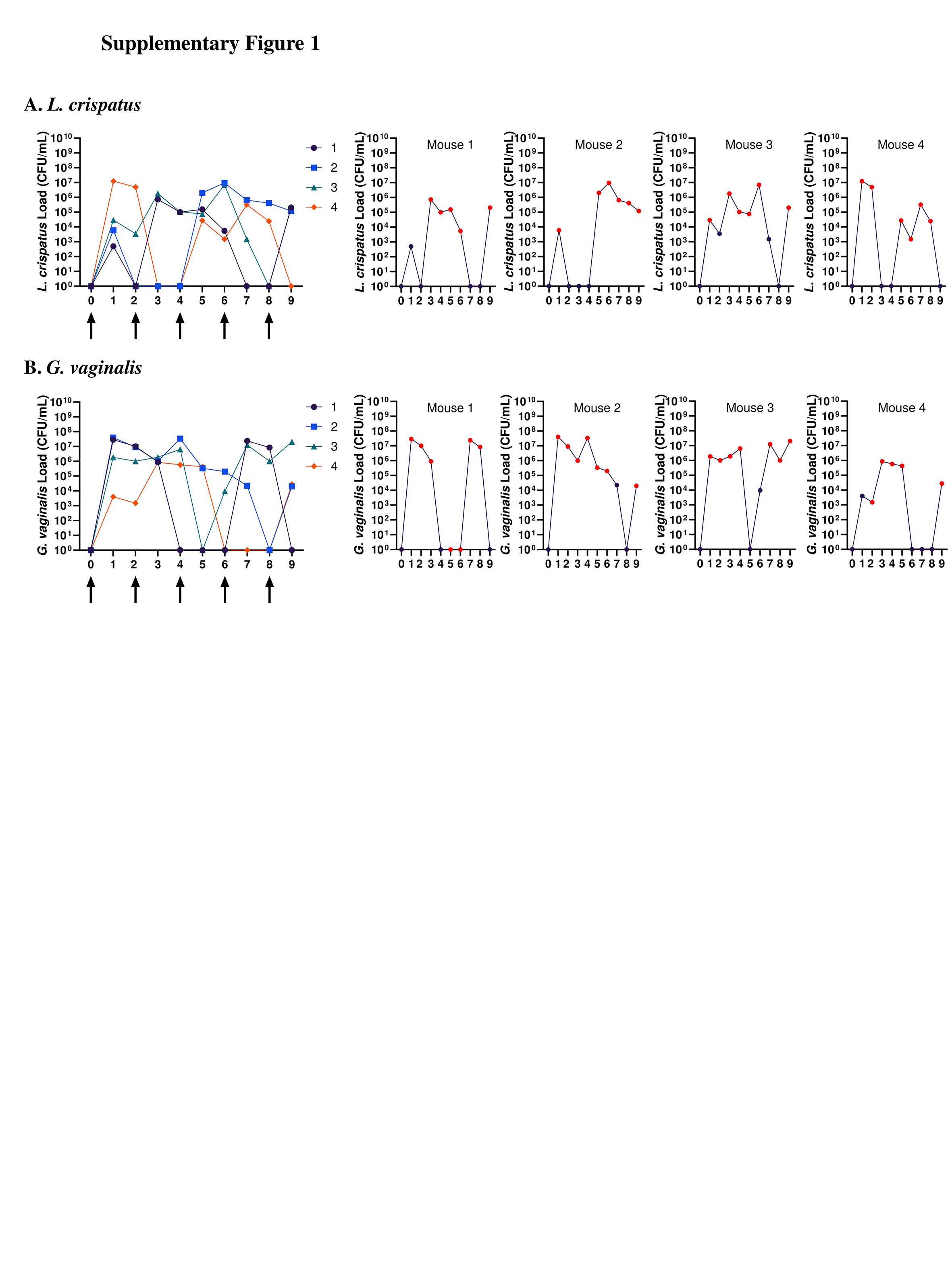

Supplement: Supplementary Figure 1 — Mice in estrus were able to stay colonized with exogenous bacteria. Female mice were inoculated five times every 48 h with 107 CFU L. crispatus or G. vaginalis. Data are from n = 4 per group from one independent experiment. Vaginal washes were collected every day until 24 h after the fifth inoculation (day 9) and assessed using quantitative plating assays. Bacterial colonies of the inoculated species types were counted in L. crispatus inoculated (A) and G. vaginalis inoculated (B) mice. Different coloured points denote different mice. Individual mouse data is plotted beside the cumulative graphs. Red dots in these graphs denote the mouse was in estrus and black dots denote the mouse was in diestrus on that day. The arrows indicate the day mice were inoculated with bacteria. The data was analyzed using a two-way ANOVA with Tukey’s multiple comparisons, but no significance was found. [file Image1.tif]

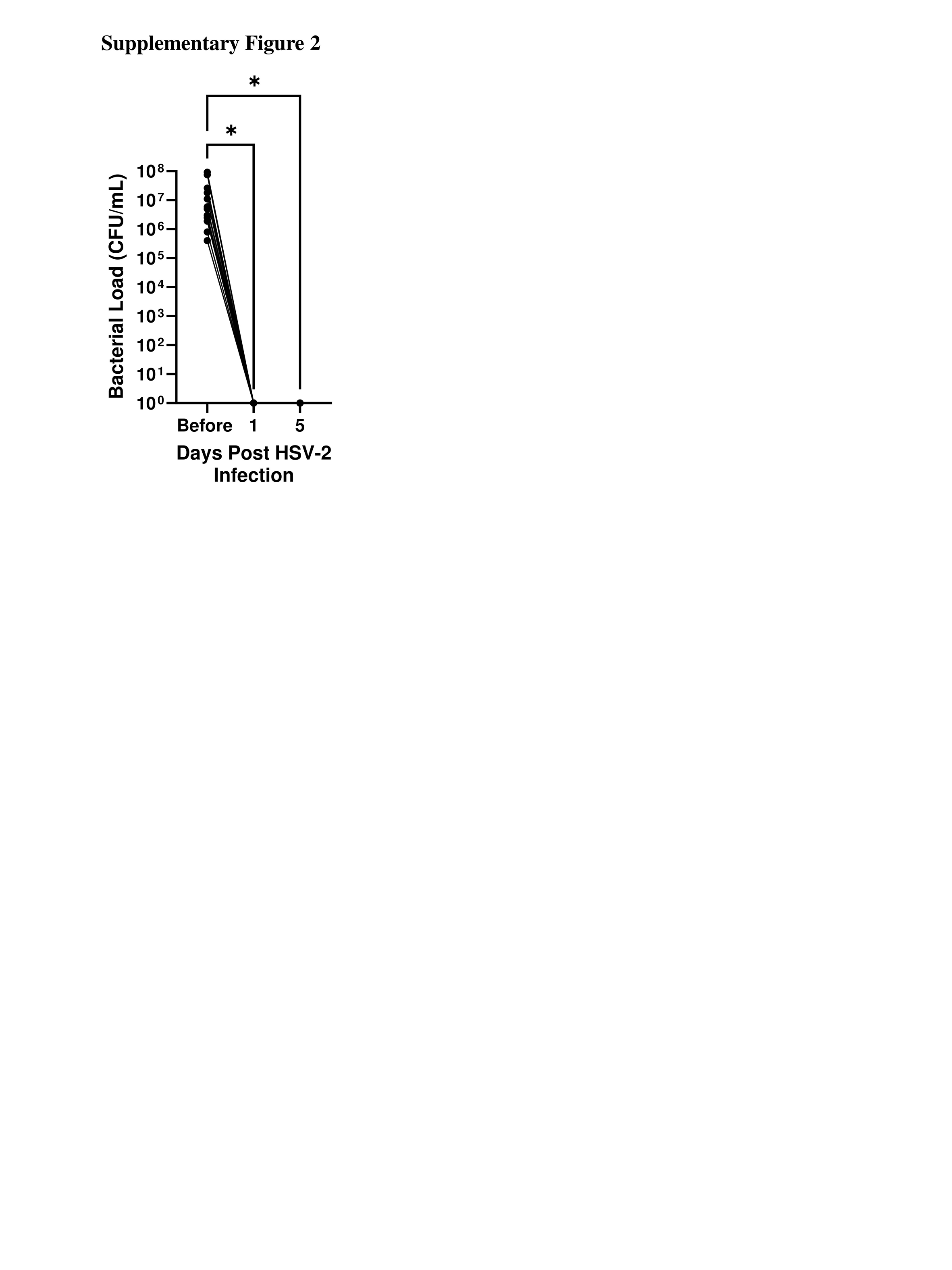

Supplement: Supplementary Figure 2 — No bacteria was detected in the vaginal tract following HSV-2 infection. Female mice were inoculated five times every 48 h with 107 CFU L. crispatus, G. vaginalis, or PBS as a no-exogenous bacteria negative control. Vaginal washes were collected 24 h before intravaginal infection with 105 PFU wildtype HSV-2, as well as one- and five-days post infection. Bacterial load was assessed using quantitative plating assays. Bacterial colonies were counted in n=14 mice and plotted. Different dots denote different mice. The data was analyzed with a one-way ANOVA with Tukey’s multiple comparisons (*p<0.05). [file Image2.tif]

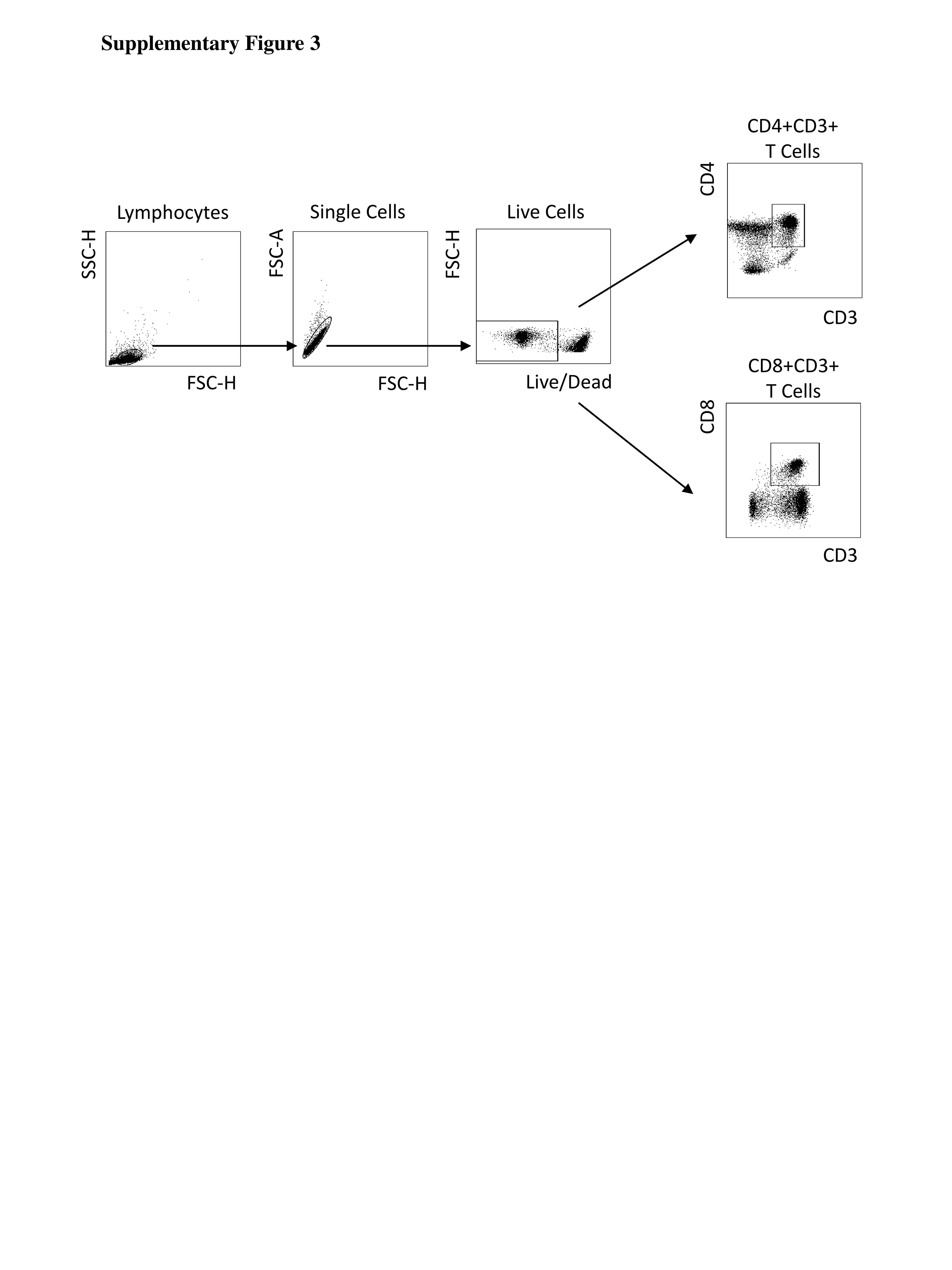

Supplement: Supplementary Figure 3 — Gating Strategy for Flow Cytometry. Female mice were intravaginally inoculated with 107 CFU L. crispatus, G. vaginalis, or PBS as a no-exogenous bacteria-negative control every 48 h for 10 days. On day 10 of the experiment, the vaginal tissue was collected, processed, and stimulated for 16 h. Cells were stained for Live/Dead staining, CD3, CD4, and CD8 (other markers for tissue-resident and functional T cells were also included). The gating strategy up to CD4+CD3+ and CD8+CD3+ T cells is depicted above. [file Image3.tif]

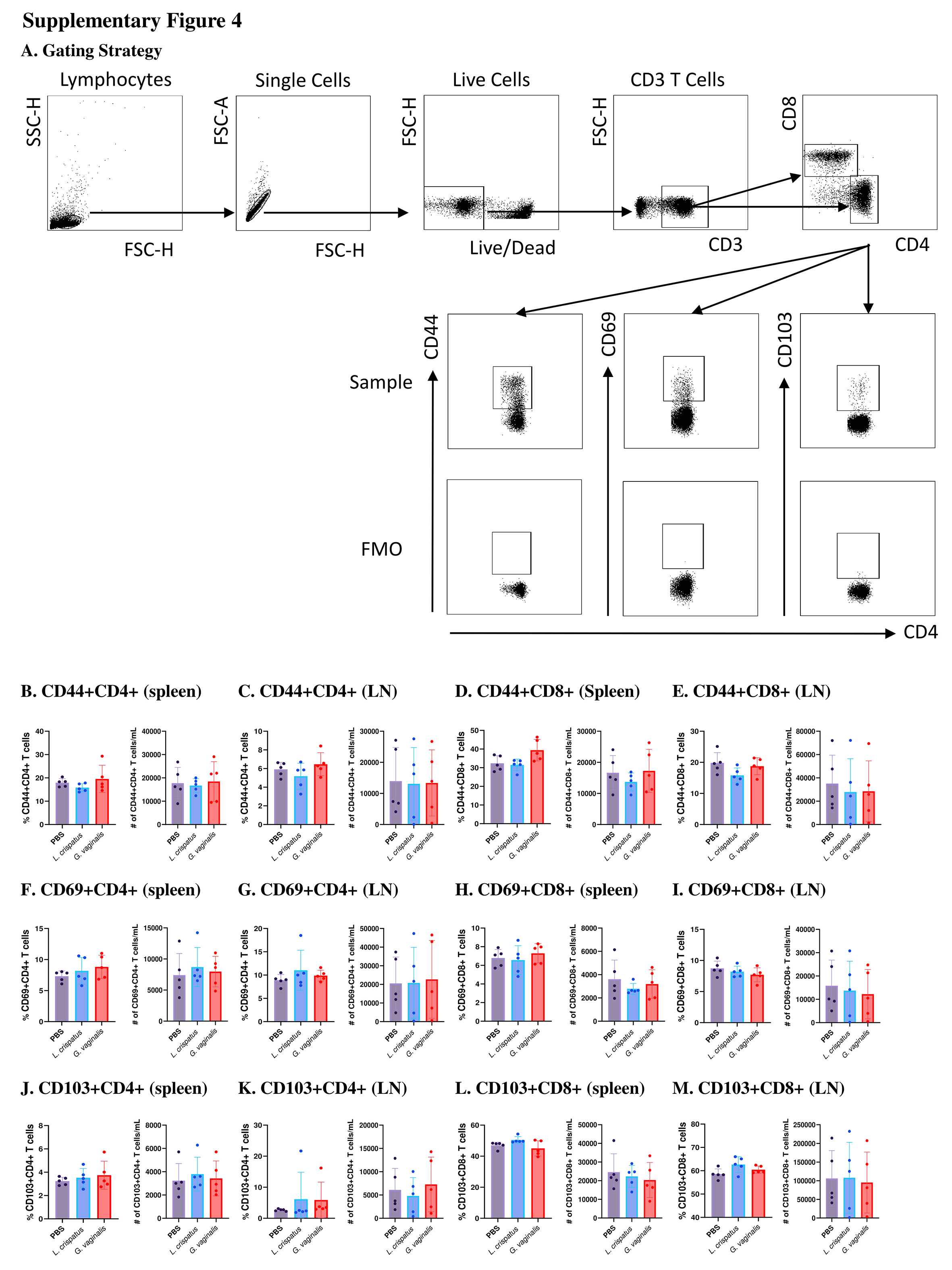

Supplement: Supplementary Figure 4 — There were no differences in T cell populations in the spleen and lymph nodes of VMB-inoculated mice. Female mice were intravaginally inoculated with 107 CFU, L. crispatus, G. vaginalis, or PBS as a no-exogenous bacteria-negative control every 48 h for 10 days. On day 10 of the experiment, the spleen and iliac lymph nodes (LN) were collected, processed, and stimulated for 16 h. Cells were stained for Live/Dead staining, CD3, CD4, CD8, CD44, CD103, and CD69, ran on the Cytoflex flow cytometer, and analyzed using FloJo software. The gating strategy is shown in panel (A). The percent population and absolute count of CD44+CD4+ T cells in the spleen (B) and LN (C), CD44+CD8+ T cells in the spleen (D) and LN (E), CD69+CD4+ T cells in the spleen (F) and LN (G), CD69+CD8+ T cells in the spleen (H) and LN (I), CD103+CD4+ T cells in the spleen (J) and LN (K), and CD103+CD8+ T cells in the spleen (L) and LN (M), are depicted above. Data are from n=7 per group, from one experiment representing three independent experiments. Data was analyzed using a one-way ANOVA with Tukey’s multiple comparisons, but no significance was found. [file Image4.tif]

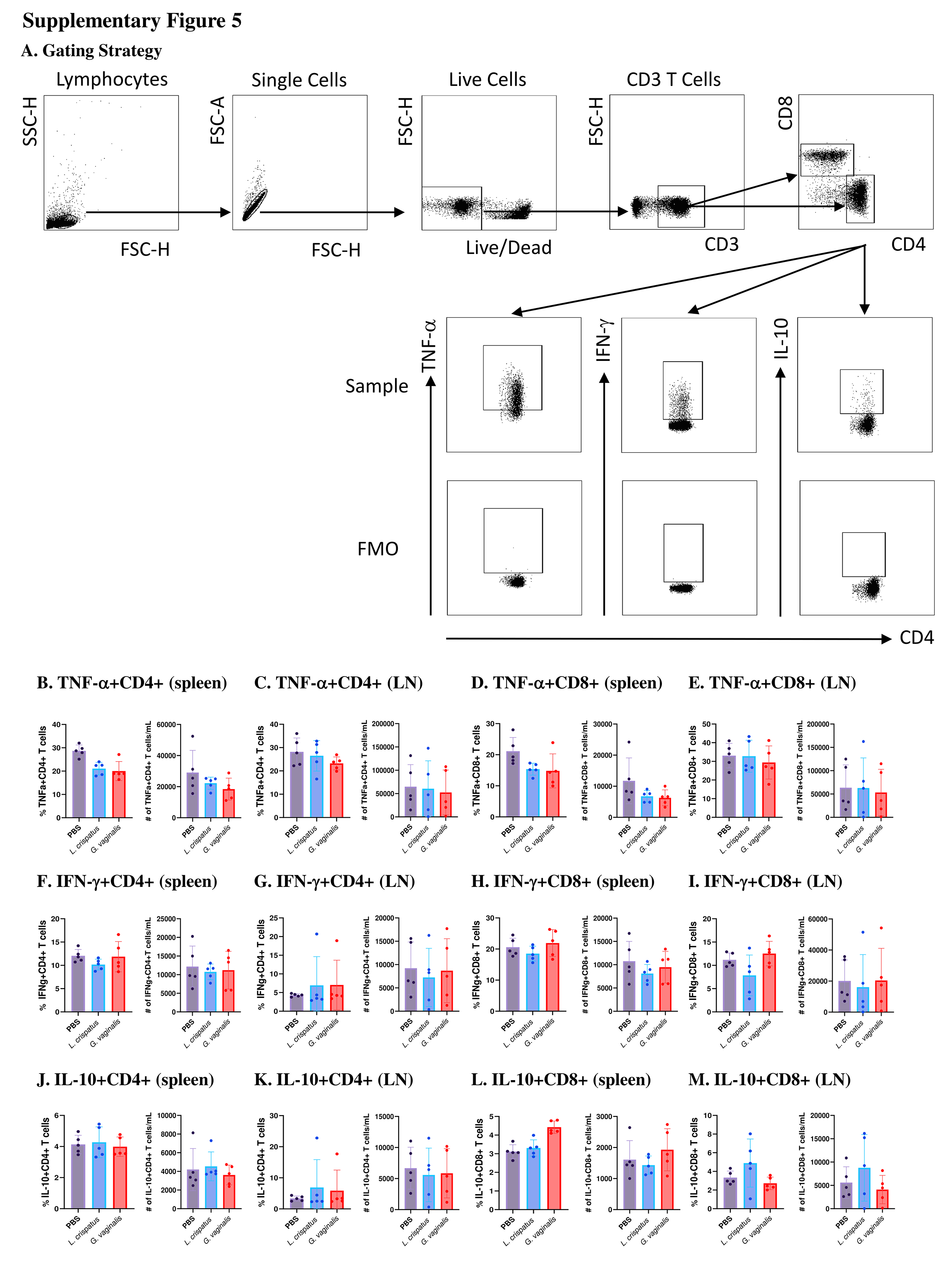

Supplement: Supplementary Figure 5 — There were no differences in cytokine-producing T cells in the spleen and lymph nodes of VMB-inoculated mice. Female mice were intravaginally inoculated with 107 CFU L. crispatus, G. vaginalis, or PBS as a no-exogenous bacteria-negative control every 48 h for 10 days. On day 10 of the experiment, the spleen and iliac lymph nodes (LN) were collected, processed, and stimulated for 16 h. Cells were stained for Live/Dead staining, CD3, CD4, CD8, TNF-α, IFN-γ, and IL-10, ran on the Cytoflex flow cytometer, and analyzed using FloJo software. The gating strategy is shown in panel (A). The percent population and absolute count of TNF-α+CD4+ T cells in the spleen (B) and LN (C), TNF-α+CD8+ T cells in the spleen (D) and LN (E), IFN-γ+CD4+ T cells in the spleen (F) and LN (G), IFN-γ+CD8+ T cells in the spleen (H) and LN (I), IL-10+CD4+ T cells in the spleen (J) and LN (K), and IL-10+CD8+ T cells in the spleen (L) and LN (M), are depicted above. Data are from n=7 per group, from one experiment representing three independent experiments. Data was analyzed using a one-way ANOVA with Tukey’s multiple comparisons, but no significance was found. [file Image5.tif]
